# Supplementary material for: Rapid synchronous type 1 IFN and virus-specific T cell responses characterize first wave non-severe SARS-CoV-2 infections
Source: Cell Rep Med. 2022 Mar 4;3(3):100557. doi: 10.1016/j.xcrm.2022.100557 (PMC8895494; doi:10.1016/j.xcrm.2022.100557)
Supplement: Document S1. Figures S1–S7 and Tables S1–S3 [file mmc1.pdf]

Cell Reports Medicine, Volume *3*

Supplemental information

Rapid synchronous type 1 IFN

and virus-specific T cell responses characterize

first wave non-severe SARS-CoV-2 infections

Aneesh Chandran, Joshua Rosenheim, Gayathri Nageswaran, Leo Swadling, Gabriele Pollara, Rishi K. Gupta, Alice R. Burton, José Afonso Guerra-Assunção, Annemarie Woolston, Tahel Ronel, Corinna Pade, Joseph M. Gibbons, Blanca Sanz-Magallon Duque De Estrada, Marc Robert de Massy, Matthew Whelan, Amanda Semper, Tim Brooks, Daniel M. Altmann, Rosemary J. Boyton, Áine McKnight, Gabriella Captur, Charlotte Manisty, Thomas Alexander Treibel, James C. Moon, Gillian S. Tomlinson, Mala K. Maini, Benjamin M. Chain, Mahdad Noursadeghi, and COVIDsortium Investigators

Supplementary materials

Contents

Supplementary Tables 2

Table S1. Baseline characteristics of the study cohort, related to Figure 1 and STAR methods. 2

Table S2. Flow cytometry detection of lymphocyte subsets, related to Figure 3, Figure 5 and flow cytometry analysis in STAR methods. 3

Table S3: Genes comprising the T cell proliferation module derived from PPD stimulated PBMC, related to derivation of a T cell proliferation in module in STAR methods and Figure S5 4

Supplementary Figures 5

Figure S1. Study consort diagram, related to Figure 1 and to sample selection in case-control study design in STAR methods. 5

Figure S2. Batch correction, molecular degree of perturbation and identification of co-regulated modules in blood transcriptomic data, related to analysis of Blood RNA sequencing data in STAR methods, and Figure 1. 6

Figure S3. Bioinformatic analysis of modules derived from upstream regulator analysis of differentially expressed genes in blood transcriptome associated with SARS-CoV-2 infection, related to Figure 1, Figure 2 and Blood RNA sequencing analysis in STAR methods. 7

Figure S4. STAT1 and CCND1 regulated module expression over time in blood transcriptomic data from individuals experimentally infected with acute respiratory viruses, related to Figure 2. 8

Figure S5. Multiparametric flow cytometry of PBMC and frequency of selected lymphocytes and their activation/proliferative state, related to Figure 3. 9

Figure S6. Identification of expanded TCRs, expansion of TCR beta chain sequences and abundance of MAIT/iNKT cell sequences in SARS-CoV-2 infection, related to Figure 4 and analysis of blood TCR sequencing data in STAR methods. 10

Figure S7. Ex vivo T cell responses to structural and non-structural peptides 16 weeks post-infection, related to Figure 4 and analysis of blood TCR sequencing data in STAR methods. 11

Supplementary Tables

Table S1. Baseline characteristics of the study cohort, related to Figure 1 and STAR methods.

**Characteristic**	**Overall, N = 96**	**Cases, N = 41[1]**	**Controls, N = 55[1]**
Age	36 (27, 47)	36 (28, 46)	36 (26, 50)
Gender (self reported)			
Female	69 (72%)	28 (68%)	41 (75%)
Male	27 (28%)	13 (32%)	14 (25%)
Ethnicity			
White	66 (69%)	25 (62%)	41 (75%)
Black	6 (6·3%)	5 (12%)	1 (1·8%)
Asian	18 (19%)	9 (22%)	9 (16%)
Other	5 (5·3%)	1 (2·5%)	4 (7·3%)
Unknown	1	1	0
Samples			
1	67 (70%)	12 (29%)	55 (100%)
2	7 (7·3%)	7 (17%)	0 (0%)
3	6 (6·2%)	6 (15%)	0 (0%)
4	10 (10%)	10 (24%)	0 (0%)
5	6 (6·2%)	6 (15%)	0 (0%)
Case-defining symptoms	36 (38%)	31 (76%)	5 (9·1%)

[1]Statistics presented: median (IQR); n (%)

Table S2. Flow cytometry detection of lymphocyte subsets, related to Figure 3, Figure 5 and flow cytometry analysis in STAR methods.

**Cell types**		**Flow cytometry staining characteristics**
T cells	CD4	CD3+, CD56-, CD4+
	CD8	CD3+, CD56-, CD8+
	Naïve	CD3+, CD45RA+, CCR7+
	TCM	CD3+, CD45RA-, CCR7+
	TEM	CD3+, CD45RA-, CCR7-
	TEMRA	CD3+, CD45RA+, CCR7-
NK cells		CD3-, CD56+
NK T cells		CD3+, CD56+
MAIT cells		CD161++, TCR Va7.2+
B cells	Naïve B cells:	CD20+, CD19+, IgD+, CD27-, CD21+ CD24+ CD38+
	Classical memory:	CD20+, CD19+, IgD-, CD27+, CD21+, (CD38+/lo, CD24+)
	Activated memory:	CD20+, CD19+, IgD-, CD27+, CD21-, (CD38-, CD24-)
	Plasma cells:	CD20-, CD19+, IgD-, CD27++, CD38++, CD138+, (CD24-,)
	Plasmablasts:	CD20-, CD19+, CD38++, CD27 +, (CD138-; CD24-)

Table S3: Genes comprising the T cell proliferation module derived from PPD stimulated PBMC, related to derivation of a T cell proliferation in module in STAR methods and Figure S5

Gene symbol	Ensembl gene ID
AURKA	ENSG00000087586
BUB1	ENSG00000169679
BUB1B	ENSG00000156970
CCNA2	ENSG00000145386
CCNB1	ENSG00000134057
CCNB2	ENSG00000157456
CDCA5	ENSG00000146670
CDCA8	ENSG00000134690
CDK1	ENSG00000170312
CDK6	ENSG00000105810
CDKN2C	ENSG00000123080
CDT1	ENSG00000167513
CENPM	ENSG00000100162
CENPN	ENSG00000166451
GINS2	ENSG00000131153
H2AC14	ENSG00000276368
H2AFX	ENSG00000188486
H3C10	ENSG00000278828
H3C12	ENSG00000197153
H3C15	ENSG00000203852
H3C4	ENSG00000197409
HIST2H3D	ENSG00000183598
KIF2C	ENSG00000142945
LMNB1	ENSG00000113368
MLF1IP	ENSG00000151725
NDC80	ENSG00000080986
PCNA	ENSG00000132646
PTTG1	ENSG00000164611
SMC4	ENSG00000113810

Supplementary Figures

Figure S1. Study consort diagram, related to Figure 1 and to sample selection in case-control study design in STAR methods.

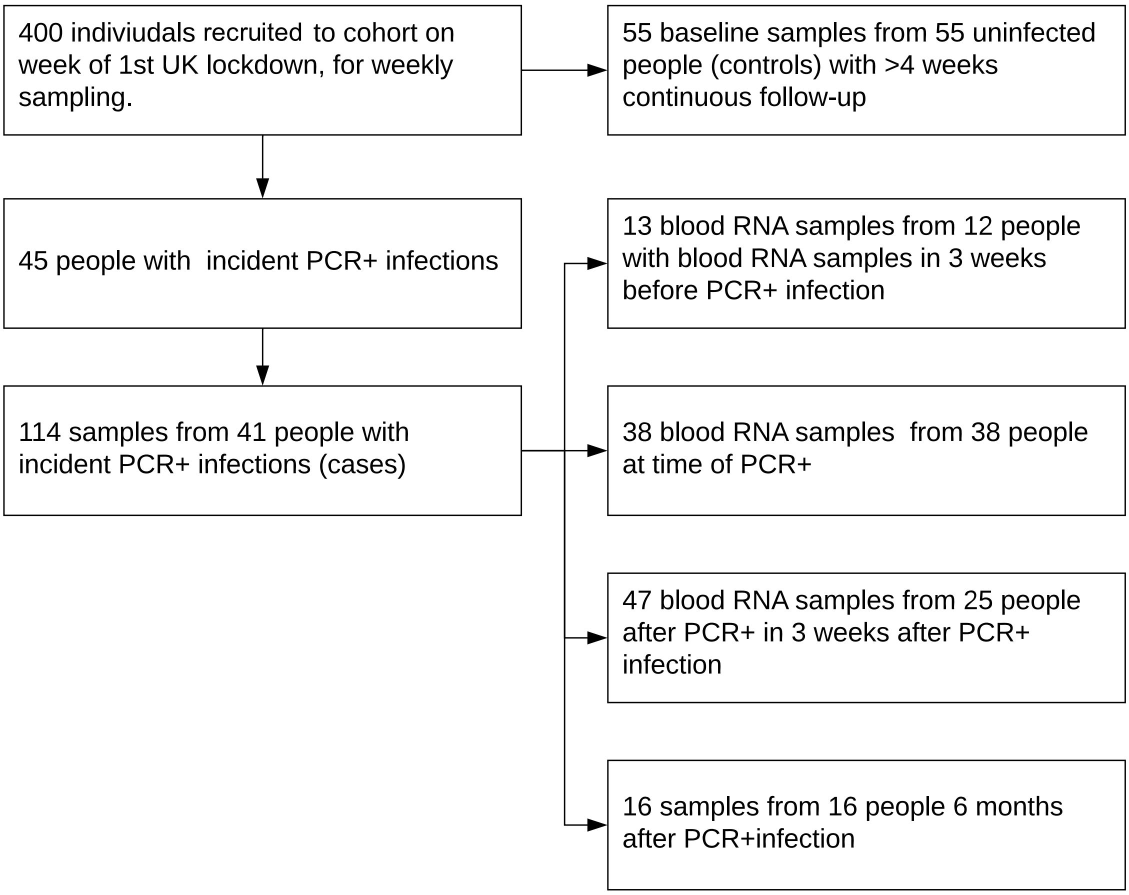

Figure S2. Batch correction, molecular degree of perturbation and identification of co-regulated modules in blood transcriptomic data, related to analysis of Blood RNA sequencing data in STAR methods, and Figure 1.

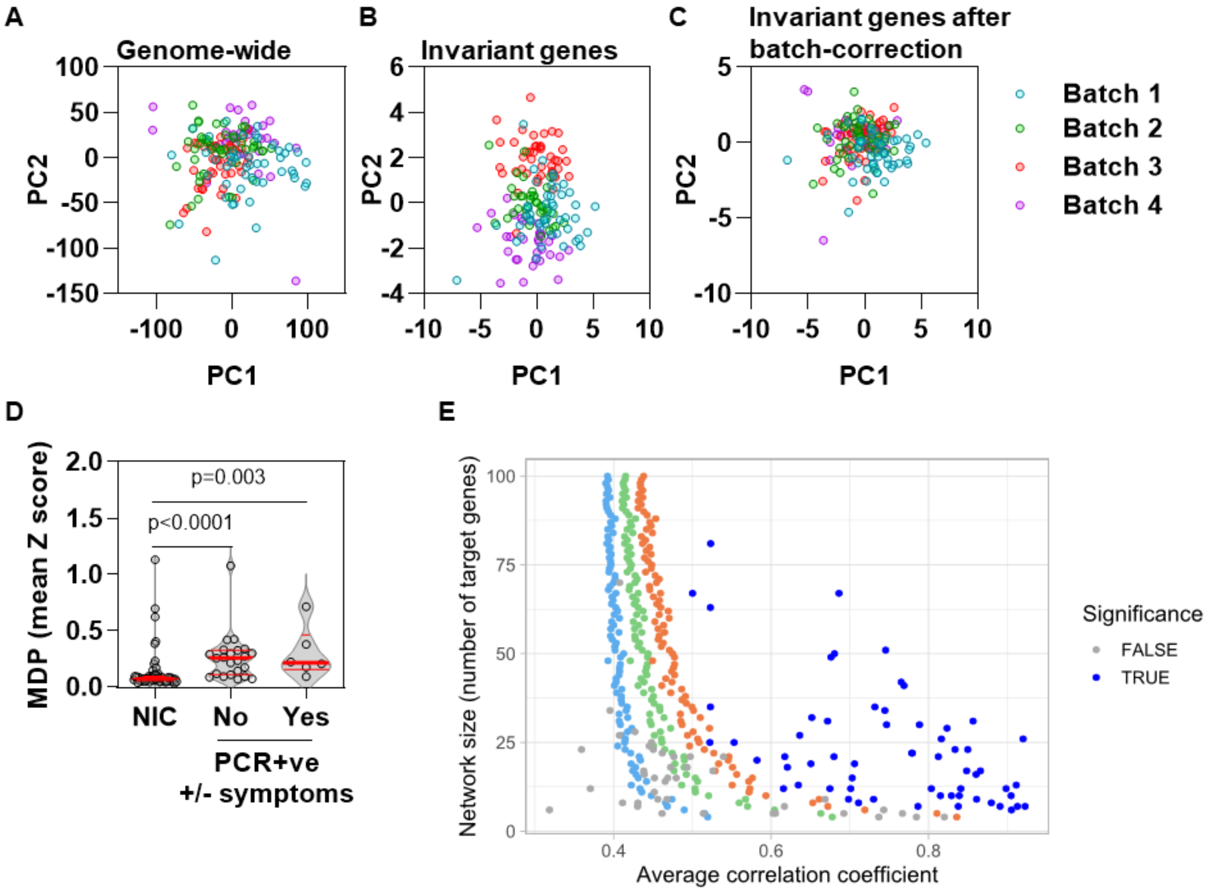

(A) Principal component analysis for genome-wide data, **(B)** 10% least variant genes from blood transcriptional profiles stratified by sample processing batch, and **(C)** for least variant genes after batch correction. **(D)** Molecular degree of perturbation (MDP) in blood transcriptomes for each individual expressed as the mean of genome-wide standard deviations (Z scores) >2 from the mean of non-infection controls (NIC), among samples from non-infection controls and samples from individuals with co-incident PCR+ve infection with (Yes) or without (No) case-defining symptoms. Individual data points shown with violin plots depicting median, IQR and frequency distributions. P values derived from Mann-Whitney tests (ns=not significant). **(E)** Figure 2, and blood RNA sequencing analysis in STAR methods. Z-scores derived from the distribution of average correlation coefficients obtained from 100 iterations of randomly selecting groups of genes from blood transcriptomic data are shown in light blue (z-score=1), green (z-score=2) and orange (z-score=3). Average correlation coefficients of molecular networks associated with upstream regulators of differentially expressed genes between individuals with incident SARS-CoV-2 infection and noninfection controls are shown in dark blue (z-score ≥2, false discovery rate ≤0.05) and grey (z-score ≤2 +/- FDR ≥0.05) compared to equivalent sized random gene networks.

Figure S3. Bioinformatic analysis of modules derived from upstream regulator analysis of differentially expressed genes in blood transcriptome associated with SARS-CoV-2 infection, related to Figure 1, Figure 2 and Blood RNA sequencing analysis in STAR methods.

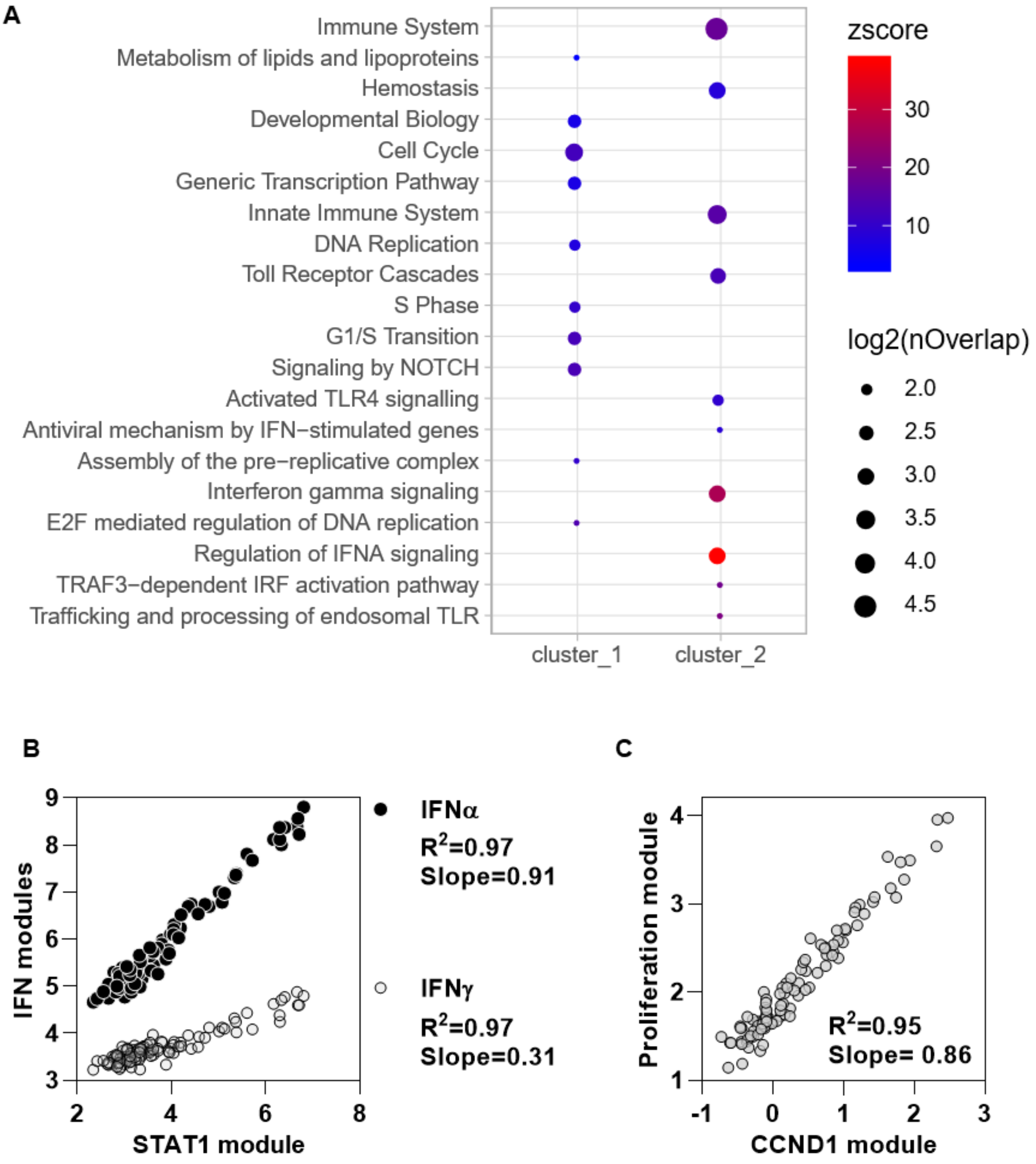

(A) Enrichment of Reactome pathways among the two clusters of predicted upstream regulators associated with co-incident infection (depicted main manuscript Figure 1 and listed Supplementary Data File 1). Node size represents number of upstream regulators associated with each pathway and statistical enrichment represented by Z score. **(B)** Correlation of STAT1-regulated transcriptional module with interferon (IFN)α and IFNγ modules, and **(C)** correlation of CCND1-regulated transcriptional module with independently derived proliferation module in all time points (-3 to +3 weeks) from individuals with SARS-CoV-2 infection.

Figure S4. STAT1 and CCND1 regulated module expression over time in blood transcriptomic data from individuals experimentally infected with acute respiratory viruses, related to Figure 2.

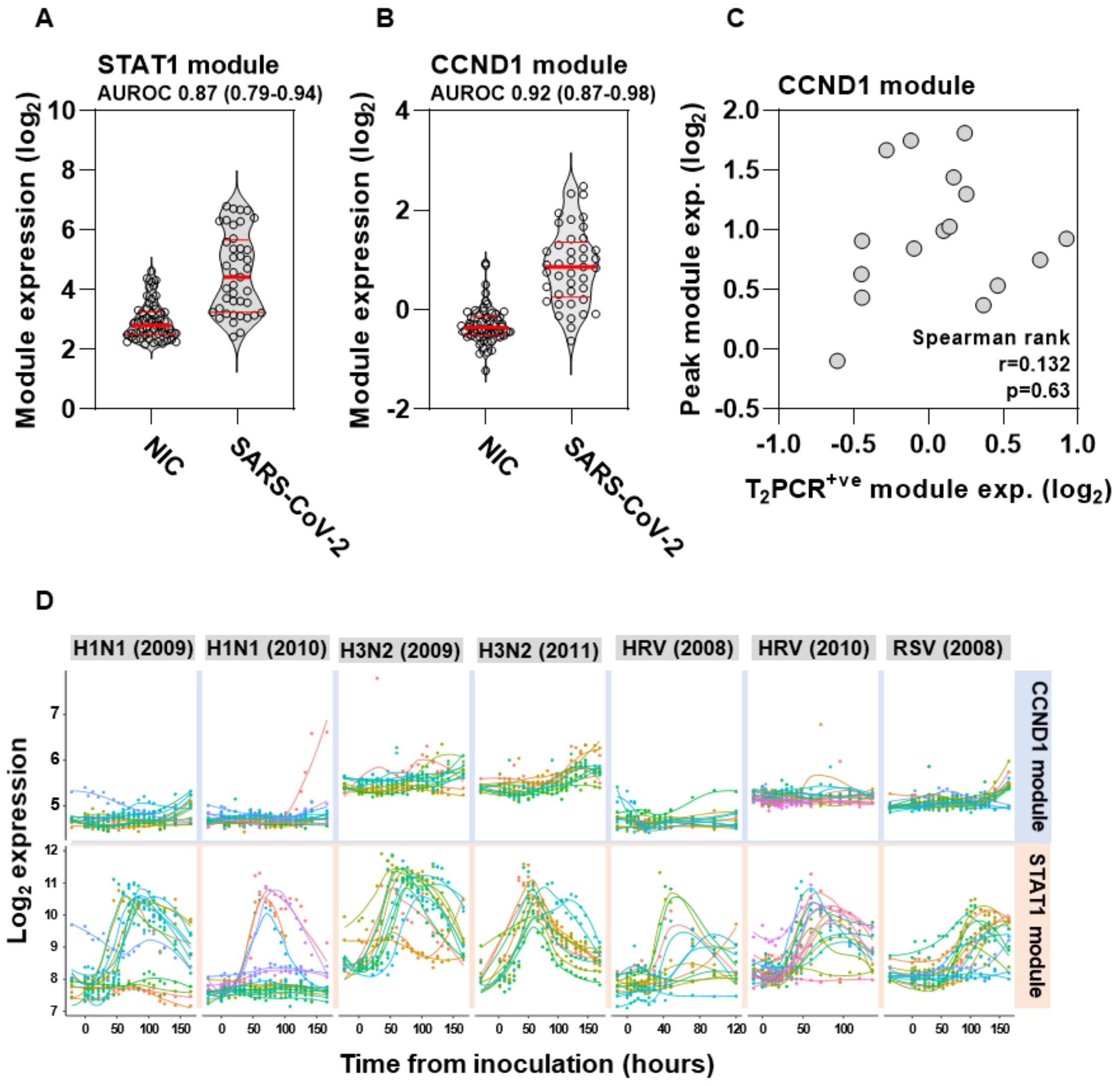

(A-B) Comparison of peak STAT-1 and CCND1-regulated module expression in samples from individuals with SARS-CoV-2 infection and non-infection controls showing area under the receiver operating curve ±95% confidence intervals for these modules to discriminate between the two groups. **(C)** Correlation of peak CCND1-regulated module expression in samples from individuals with SARS-CoV-2 infection with CCND1-regulated module expression 2 weeks after first positive PCR test. **(D)** Expression scores for STAT1 and CCND1 modules in publicly available data from seven (non-SARS-CoV-2) human viral challenge studies (GSE73072). Analysis restricted to participants with evidence of infection following inoculation, as per original study definition (total n=92 participants). Colours represent individual participants with longitudinal sampling. Each column represents a different challenge study, with the virus and study year shown in the column headers. HRV = human rhinovirus; RSV = respiratory syncytial virus.

Figure S5. Multiparametric flow cytometry of PBMC and frequency of selected lymphocytes and their activation/proliferative state, related to Figure 3.

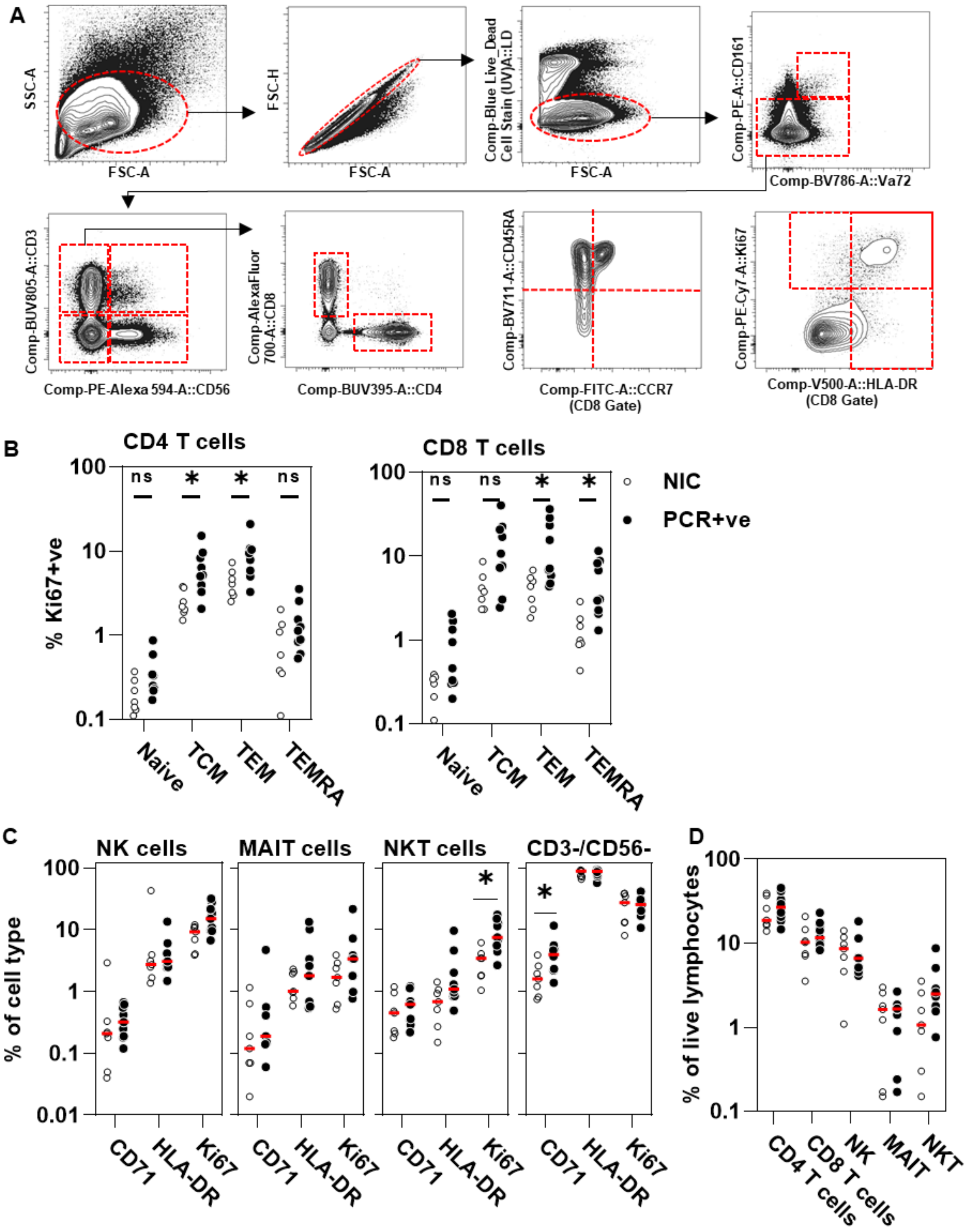

(A) Example gating strategy for flow cytometric data showing gating for; lymphocytes, singlets, live cells (fixable live dead-), MAIT cells (CD161++ TCR Va7.2+) and non-MAIT cells, CD3+CD56- T cells, CD3+CD56+ NKT cells, CD3-CD56- lymphocytes, CD3-CD56+ NK cells, CD4+ and CD8+ T cells, CD45RA vs. CCR7 co-staining, and HLA-DR vs. Ki67 co-staining. **(B)** Frequency of CD4+ and CD8+ T cell subsets, **(C)** non-T cell lymphocyte subsets expressing selected activation markers (CD71 and HLA-DR) and the cell proliferation marker (Ki67), and **(D)** comparative frequency of selected lymphocyte subsets among participants with co-incident infection (PCR+) and non-infection controls (NIC). Data points with median values (red). (*p<0.05 by Mann-Whitney Test for each group compared to NIC).

Figure S6. Identification of expanded TCRs, expansion of TCR beta chain sequences and abundance of MAIT/iNKT cell sequences in SARS-CoV-2 infection, related to Figure 4 and analysis of blood TCR sequencing data in STAR methods.

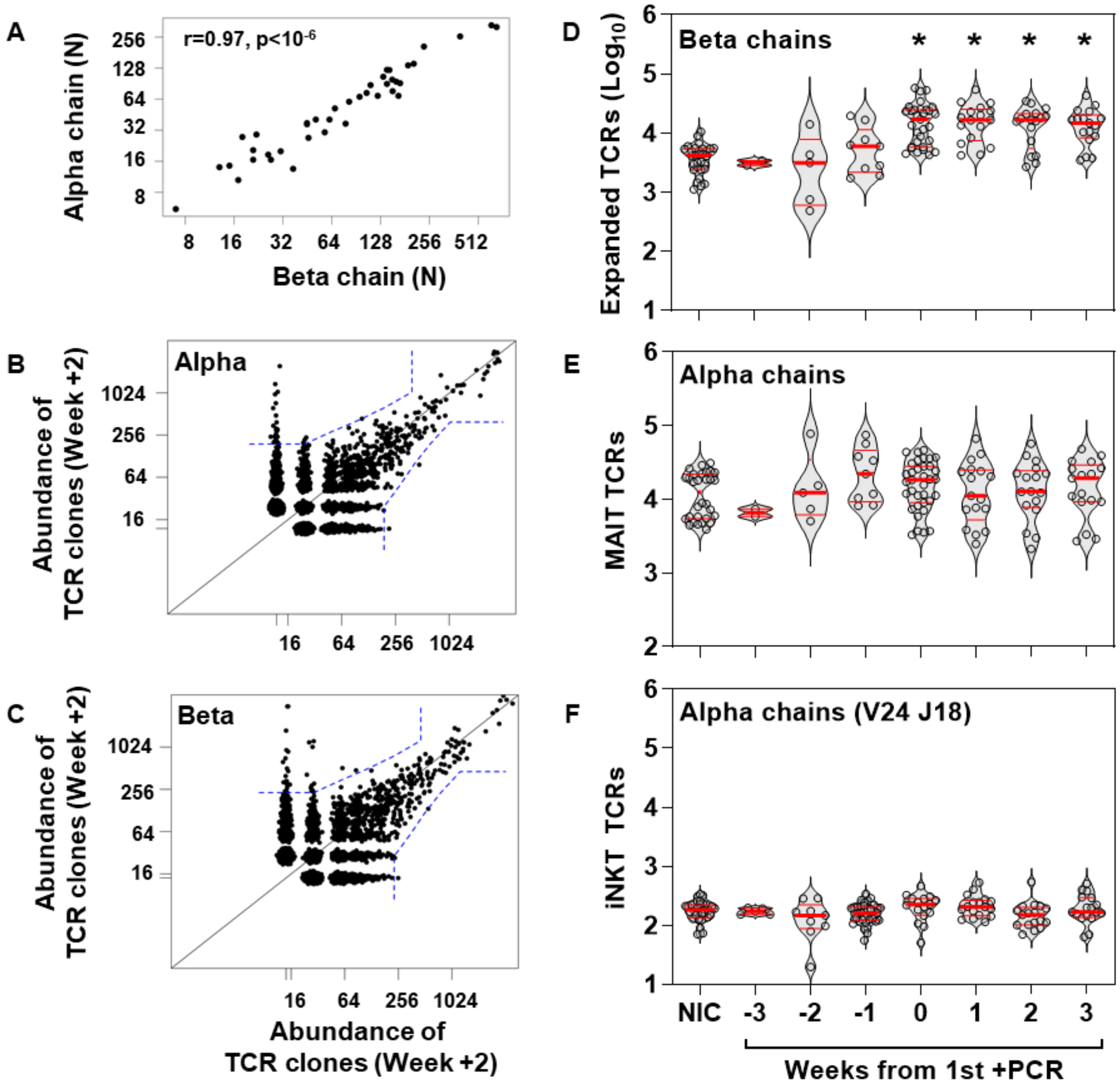

(A) Correlation of numbers of alpha and beta chain TCR sequences per sample. **(B)** Representative examples of identification of statistically expanded alpha, and beta chain TCR sequences by comparison of abundance of individual TCR sequences between two time points (blue dashed boundaries represent the thresholds for abundance of TCR sequences with false discovery rate of <1 in 1000) **(C)** Enumeration of expanded TCR alpha chain sequences (per million total sequences) in non-infection controls (NIC) and samples from infected individuals stratified by time from first positive PCR. **(D)** Abundance of MAIT and **(E)** iNKT cell associated alpha chain sequence in non-infection controls and samples from infected individuals stratified by time from first positive PCR. Individual data points shown with violin plots depicting median, IQR and frequency distributions. (*FDR<0.05 by Kruskal-Wallis Test for each group compared to NIC.

Figure S7. Ex vivo T cell responses to structural and non-structural peptides 16 weeks post-infection, related to Figure 4 and analysis of blood TCR sequencing data in STAR methods.

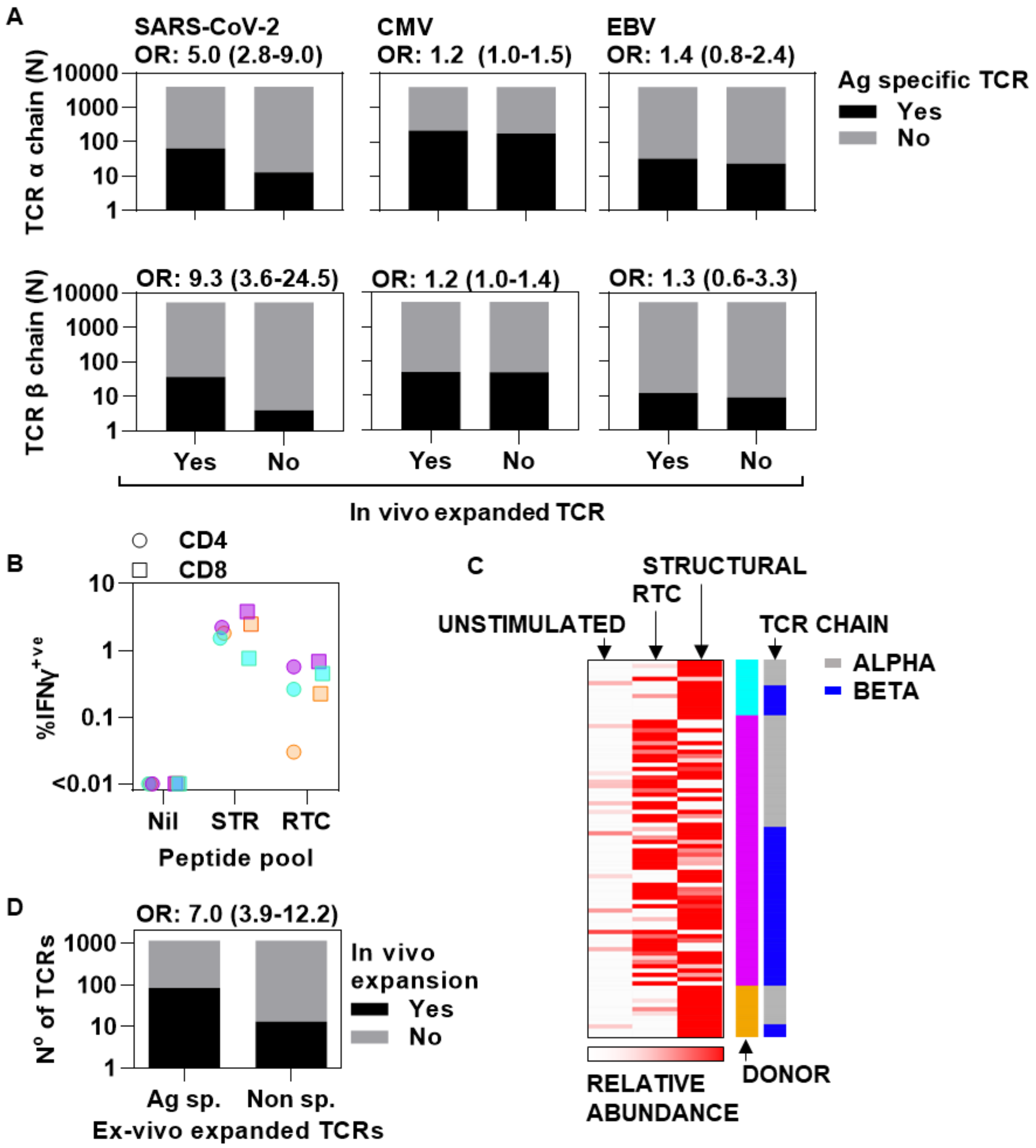

(A) Number of antigen-specific TCR sequences (alpha and beta-chains) for SARS-CoV-2, cytomegalovirus (CMV) and Epstein-Barr Virus (EBV) among expanded TCR sequences in all time points (-3 to +3 weeks) from individuals with SARS-CoV-2 infection and among non-expanded TCRs from the same samples, giving the odds ratio (±95% confidence interval, Fisher's exact test) for enrichment of antigen specific TCR sequences in each case. **(B)** Frequency of interferon (IFN)γ producing CD4 and CD8 T cells in PBMC (N=3 individuals stratified by colour of data points) following 8 days culture in the presence of IL2 and soluble anti-CD28 ±stimulation with overlapping peptides covering structural (STR) or replication and transcription complex (RTC) proteins. **(C)** Relative abundance of TCR alpha and beta chains from the experiments in (A) that coincide with expanded sequences in peripheral blood of the same individuals at the time of incident PCR positive infection. **(D)** Overlap of in vivo expanded TCR sequences (alpha and beta-chains) associated with incident PCR positive SARS-CoV-2 infection with ex vivo expanded TCRs SARS-CoV-2 peptide stimulated and unstimulated PBMC giving the odds ratio (±95% confidence interval, Fisher's exact test) for enrichment of virus specific TCR sequences.
