# Supplementary material for: Rapid synchronous type 1 IFN and virus-specific T cell responses characterize first wave non-severe SARS-CoV-2 infections
Source: Cell Rep Med. 2022 Mar 4;3(3):100557. doi: 10.1016/j.xcrm.2022.100557 (PMC8895494; doi:10.1016/j.xcrm.2022.100557)
Supplement: Document S2. Article plus supplemental information [file mmc4.pdf]

Cell Reports
Medicine

Article

Rapid synchronous type 1 IFN and virus-specific T cell responses characterize first wave non-severe SARS-CoV-2 infections

Graphical abstract

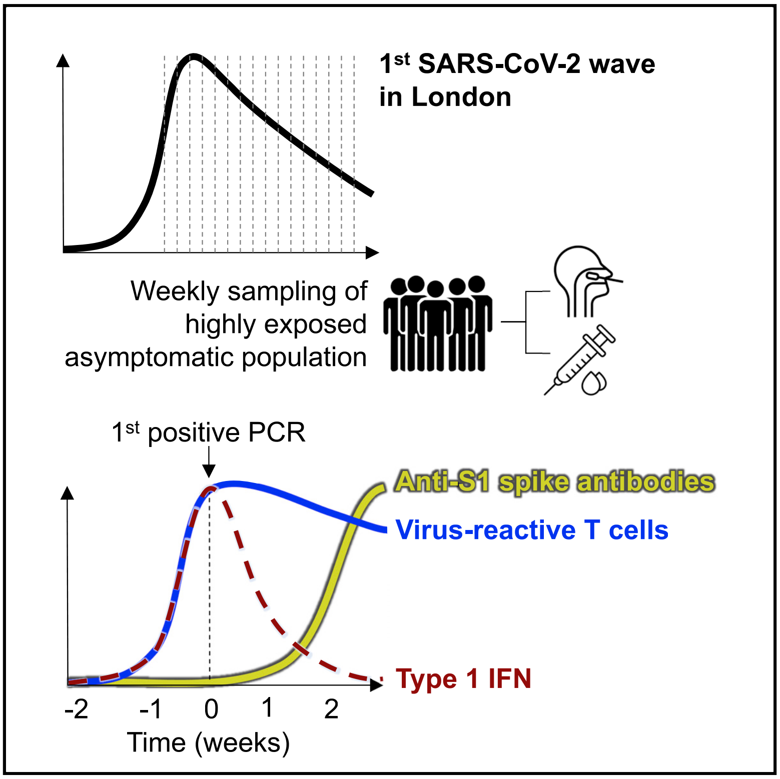

Authors

Aneesh Chandran, Joshua Rosenheim, Gayathri Nageswaran, ..., Benjamin M. Chain, Mahdad Noursadeghi, COVIDsortium Investigators

Correspondence

m.noursadeghi@ucl.ac.uk

In brief

Non-severe SARS-CoV-2 infection in unvaccinated virus-naive individuals may reveal correlates of protection against future variants. In this context, Chandran et al. show rapid type 1 interferon and virus-specific T cell responses 1–2 weeks before virus-specific antibodies, supporting a role for T cell-mediated heterologous protection in next-generation vaccines.

Highlights

- Interferon response to non-severe SARS-CoV-2 can precede PCR detection of infection
- Virus-specific T cell responses are equally rapid but independent of interferon
- Virus-specific antibodies lagged 1–2 weeks behind T cells in first wave infections

Chandran et al., 2022, Cell Reports Medicine *3*, 100557
March 15, 2022 © 2022 The Authors.
https://doi.org/10.1016/j.xcrm.2022.100557

Cell Reports Medicine

CellPress
OPEN ACCESS

Article

Rapid synchronous type 1 IFN and virus-specific T cell responses characterize first wave non-severe SARS-CoV-2 infections

Aneesh Chandran,[1] Joshua Rosenheim,[1] Gayathri Nageswaran,[1] Leo Swadling,[1] Gabriele Pollara,[1] Rishi K. Gupta,[2] Alice R. Burton,[1] José Afonso Guerra-Assunção,[1] Annemarie Woolston,[1] Tahel Ronel,[1] Corinna Pade,[3] Joseph M. Gibbons,[3] Blanca Sanz-Magallon Duque De Estrada,[1] Marc Robert de Massy,[1] Matthew Whelan,[1] Amanda Semper,[4] Tim Brooks,[4] Daniel M. Altmann,[5] Rosemary J. Boyton,[6,7] Áine McKnight,[3] Gabriella Captur,[8,9] Charlotte Manisty,[8] Thomas Alexander Treibel,[8] James C. Moon,[8] Gillian S. Tomlinson,[1] Mala K. Maini,[1] Benjamin M. Chain,[1] Mahdad Noursadeghi,[1,10,*] and COVIDsortium Investigators

[1]Division of Infection and Immunity, University College London, London WC1E 6BT, UK
[2]Institute for Global Health, University College London, London WC1E 6BT, UK
[3]Blizard Institute, Barts and the London School of Medicine and Dentistry, Queen Mary University of London, London E1 4NS, UK
[4]National Infection Service, Public Health England, Porton Down, Salisbury SP4 0JQ, UK
[5]Department of Immunology and Inflammation, Imperial College London, London SW7 2BX, UK
[6]Department of Infectious Disease, Imperial College London, London SW7 2BX, UK
[7]Lung Division, Royal Brompton and Harefield Hospitals, Guy's and St Thomas' NHS Foundation Trust, London, UK
[8]Institute of Cardiovascular Sciences, University College London, London WC1E 6BT, UK
[9]MRC Unit for Lifelong Health and Ageing, University College London, London WC1E 6BT, UK
[10]Lead contact
*Correspondence: m.noursadeghi@ucl.ac.uk
https://doi.org/10.1016/j.xcrm.2022.100557

SUMMARY

Effective control of SARS-CoV-2 infection on primary exposure may reveal correlates of protective immunity to future variants, but we lack insights into immune responses before or at the time virus is first detected. We use blood transcriptomics, multiparameter flow cytometry, and T cell receptor (TCR) sequencing spanning the time of incident non-severe infection in unvaccinated virus-naive individuals to identify rapid type 1 interferon (IFN) responses common to other acute respiratory viruses and cell proliferation responses that discriminate SARS-CoV-2 from other viruses. These peak by the time the virus is first detected and sometimes precede virus detection. Cell proliferation is most evident in CD8 T cells and associated with specific expansion of SARS-CoV-2-reactive TCRs, in contrast to virus-specific antibodies, which lag by 1–2 weeks. Our data support a protective role for early type 1 IFN and CD8 T cell responses, with implications for development of universal T cell vaccines.

INTRODUCTION

The host response in non-severe SARS-CoV-2 infection during the first epidemic wave, before vaccination, incorporates the mechanisms of effective host-defense in naive populations. These mechanisms remain a research priority because they may reveal determinants of protection against future variants of SARS-CoV-2 able to escape current vaccines, or future novel coronaviruses. To date, our knowledge has been limited to immune responses after the detection of the virus or onset of symptoms and to cross-sectional studies in which the time of infection was undefined. These data have provided strong evidence that germline encoded innate immunity mediated by type 1 interferon (IFN) responses contribute to protection against critical illness,[1–3] although the extent to which they abrogate early symptomatic disease is not known. Perhaps more importantly, we still have very limited insights into the potential role of adaptive immune responses in early protection against disease in the first epidemic wave.[4] These are of particular interest because they may inform the design of the next generation of vaccines with potential to provide cross-reactive immunity to future variants. We recently reported T cell responses in a subset of individuals associated with evidence for very early termination of infection before detectable viral replication or even seroconversion.[5] The extent to which adaptive immune responses are a general feature of early responses in non-severe disease is not known. We sought to describe the temporal kinetics and relationships between the earliest immune responses to infection with an unbiased systems-level approach using genome-wide transcriptional profiling of weekly blood samples before, during, and after incident SARS-CoV-2 infections during the first epidemic wave in London and compared our findings with responses to other acute respiratory viruses using publicly available data from human challenge experiments.

This is an open access article under the CC BY license (http://creativecommons.org/licenses/by/4.0/).

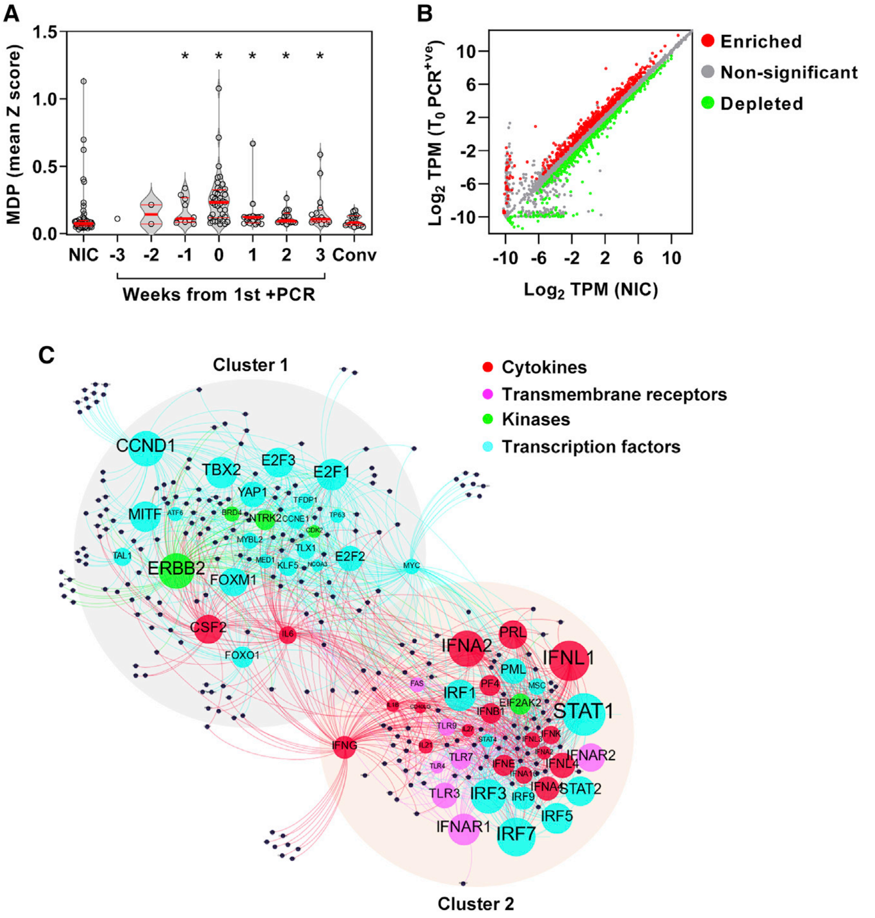

Figure 1. Incident SARS-CoV-2 infection associated with perturbation of blood transcriptome reflecting type 1 IFN and cell proliferation responses

(A) Molecular degree of perturbation (MDP) in blood transcriptomes for each individual expressed as the mean of genome-wide standard deviations (*Z* scores) from the mean of non-infection controls (NICs). Among NICs, individuals with incident infection are stratified by weeks from first positive PCR and convalescent samples 5–6 months after incident infection. Individual data points are shown with violin plots depicting median, IQR, and frequency distributions (*FDR < 0.05 by Kruskal-Wallis test for each group compared with NIC).

(B) Differentially expressed genes in blood transcriptomes at time of first positive PCR (T_0_PCR^{+ve}) compared with NICs. (TPM, transcripts per million).

(C) Predicted upstream regulators (labeled nodes) stratified by molecular function for differentially expressed genes (black nodes). Size of the nodes for upstream regulators is proportional to -Log10 p value. Nodes were clustered using Force Atlas 2 algorithm in GEPHI (version 0.9.2).

RESULTS

Type 1 IFN and cell proliferation responses from 1 week before to 3 weeks after detection of asymptomatic and mild SARS-CoV-2 infection

We undertook a nested case-control study derived from a cohort of 400 healthcare workers at one London hospital recruited from March 23, 2020 to undergo weekly nasopharyngeal swab PCR tests and blood sampling when fit to attend work, as described previously.[6–10] In this cohort, we detected 45 incident infections by PCR. Among these cases, we obtained 114 blood transcriptional profiles from 41 individuals spanning 3 weeks before to 3 weeks after the first PCR-positive result, including 12 individuals for whom samples were available before the first positive PCR. We also profiled convalescent samples from 16/41 individuals 5–6 months later. We compared these data to blood transcriptional profiles obtained from baseline samples in 55 consecutive uninfected controls who remained PCR- and seronegative for SARS-CoV-2 during follow-up (Figure S1; Table S1). None of the individuals who became infected required hospitalization. Among 38 individuals for whom blood transcriptomic data were available at the time of first positive PCR, 29 had no contemporary symptoms attributable to SARS-CoV-2 infection. Genome-wide transcriptional profiles from those who experienced an infection showed greatest perturbation compared with uninfected controls, both at the time of the first positive PCR test and independent of symptoms (Figure 1A; Figure S2D). Their profiles were significantly different from uninfected controls from the week before the first positive PCR to 3 weeks afterward. Six-month convalescent samples from a subset of these individuals were not significantly different from uninfected controls, indicating that the blood transcriptome had fully reverted to the baseline.

To investigate the host response to infection, we identified differentially expressed transcripts by comparison of profiles from the time of first positive viral PCR to those of uninfected controls (Figure 1B). These were subjected to upstream regulator enrichment analysis to identify molecular pathways predicted to be activated at the level of cytokines, transmembrane receptors, kinases, and transcription factors that may be responsible for differential gene expression (Data S1). We filtered groups of target genes associated with each upstream regulator to include only those that had significantly greater co-correlated expression than would be expected at random in our blood transcriptomes to increase our confidence that these represent co-regulated genes in each molecular pathway (Figure S2E). Among those that were retained, the associated upstream regulators formed two clusters resulting from overlapping associations with target genes (Figure 1C), reflecting two predominant biological pathways. These were type 1 IFN responses and cell-cycle activity, as surrogates for innate immune activation and cellular proliferation, respectively (Figure S3A). We collated the differentially expressed genes linked to the most statistically enriched upstream regulator in each of the two clusters as a transcriptional module, resulting in a signal transducer and activator of transcription (STAT)1-regulated module to represent type 1 IFN responses and a Cyclin D1 (CCND1)-regulated module to

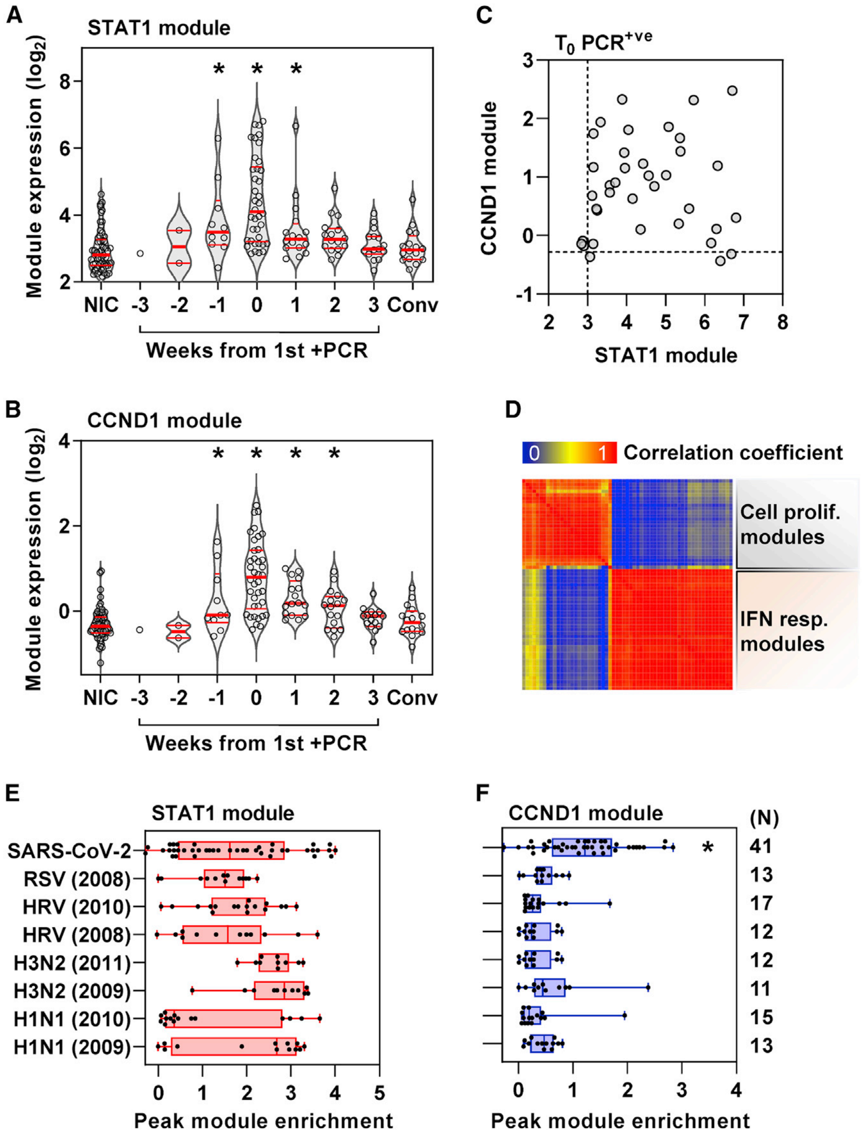

Figure 2. Cell proliferation response discriminates SARS-CoV-2 infection from other acute viruses and is not correlated with type 1 IFN response

(A and B) (A) Expression of STAT1 module (representative of type 1 IFN response) and (B) CCND1 module (representative of cell proliferation response) in blood transcriptomic data stratified by time to first positive SARS-CoV-2 infection, compared with NICs and convalescent samples 5–6 months after incident infection. Individual data points are shown with violin plots depicting median, IQR, and frequency distributions (*FDR < 0.05 by Kruskal-Wallis test for each group compared with NIC).

(C and D) (C) Comparison of STAT1 and CCND1 module expression at time of first positive PCR (dashed lines represent the upper limit of the 95% CI of median of NICs) and (D) co-correlation matrix between all type 1 IFN and cell proliferation modules at time of first positive PCR.

(E and F) Comparison of (E) STAT1 and (F) CCND1 module expression associated with co-incident SARS-CoV-2 infection compared with peak expression of these modules in experimental human challenge infections using respiratory syncytial virus (RSV), human rhinovirus (HRV), or influenza virus (H3N2 and H1N1), stratified by different datasets indicated by year (*FDR < 0.05 by Kruskal-Wallis test in SARS-CoV-2 infection compared with all other groups).

represent the cell proliferation response. The validity of the functional annotation for each of these modules was confirmed by investigating their correlation and covariance with independently derived transcriptional signatures for type 1 and type 2 IFN responses and for cell proliferation. The STAT1 module correlated with both IFN response modules but showed much greater covariance with the type 1 IFN signatures (Figure S3B), consistent with our bioinformatic analysis of the functional pathway represented by this cluster of differentially expressed genes. Similarly, we found that the CCND1-regulated gene expression module showed good correlation and covariance with an independently derived cell proliferation module (Figure S3C). Type 1 IFN and cell proliferation responses both peaked with co-incident infection (Figures 2A and 2B), but significant increases in these responses were also evident in the week before the first positive PCR result. Type 1 IFN responses remained significantly elevated for 1 week after the first positive PCR, whereas the cell proliferation response remained elevated for 2 weeks after the first positive PCR. The peak of each of these responses over this time course discriminated infected individuals from non-infected controls with area under the receiver operating characteristic curve (AUROC) of 0.87 (95% CI, 0.78–0.94) and 0.92 (95% CI, 0.87–0.98) for the STAT1- and CCND1-regulated modules, respectively (Figures S4A and S4B), giving a measure of the consistency of both these responses in infected individuals. Despite this and the overlap in the temporal profiles of these two responses, the enrichment of STAT1- and CCND1-regulated modules representing each response at the individual participant level did not correlate, suggesting that they may be independently regulated or subject to idiosyncratic capacity for each of these responses at the level of individual participants (Figure 2C). The same observation was evident for differentially expressed genes combined as modules associated with each of the upstream regulators that reflected type 1 IFN or cell proliferation modules (Figure 2D).

Cell proliferation responses distinguish SARS-CoV-2 infection from other acute respiratory viruses and are predominantly attributable to T cell responses

Next, we compared the type 1 IFN and cell proliferation responses to incident SARS-CoV-2 infection with those of other acute respiratory viruses by comparing the peak expression of the STAT1- and CCND1-regulated modules in our cohort to that of publicly

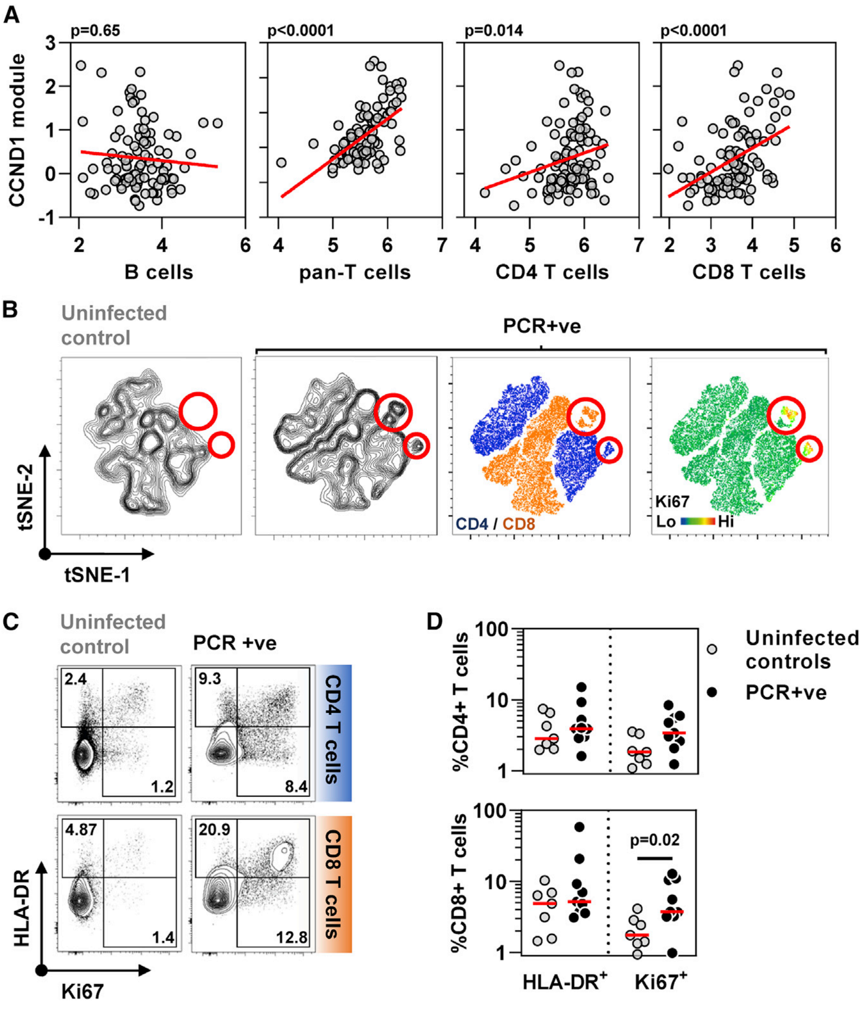

Figure 3. Cell proliferation response to SARS-CoV-2 infection in blood transcriptomic data is attributable to T cell proliferation

(A) Correlation of CCND1 module (representative of cell proliferation response) in all time points (−3 to +3 weeks) from individuals with SARS-CoV-2 infection with each of blood transcriptomic modules representative of B cells, pan-T cells, CD4 T cells, and CD8 T cells (regression lines shown in red, p values for Spearman rank correlations).

(B) tSNE plots of T cells from non-infected controls or individuals with co-incident PCR-positive SARS-CoV-2 infection. Contour plots are shown in the two left-hand panels, followed by dot plots colored by CD4/CD8 staining or relative Ki67 staining as a proliferation marker. Red circles highlight a population of Ki67 high CD4 and CD8 cells exclusive to the PCR+ group (tSNE derived from flow cytometry data of CD4 and CD8 T cells, seven NICs, and nine PCR-positive).

(C) Representative flow cytometry data for HLA-DR and Ki67 staining in either CD4 T cells or CD8 T cells from one non-infected control and one SARS-CoV-2 infected individual at the time of the first positive PCR infection. Numbers indicate percent positive for each marker including double-positives.

(D) Summary HLA-DR and Ki67 staining data and median from seven uninfected controls and nine individuals with co-incident infection in either CD4 T cells or CD8 T cells. p value shown for Mann-Whitney test.

available longitudinal blood transcriptomic data derived from human challenge experiments with respiratory syncytial virus, human rhinovirus, and influenza virus (Figure S4D).[11] Comparable enrichment of the type 1 IFN response was evident in each of these infections (Figure 2E), but the cell proliferation response was significantly greater to SARS-CoV-2 than the peak response to any of the other acute respiratory virus infections (Figure 2F). Of note, the peak cell proliferation response to SARS-CoV-2 infection did not correlate with the persistence of this response represented by CCND1 module expression at 2 weeks after the first positive PCR (Figure S4C). We tested the hypothesis that the cellular proliferation response may arise from rapid B cell or T cell expansion in response to infection by evaluating the correlation between the CCND1 module and expression of validated cell-type-specific signatures (Figure 3A). CCND1 module expression correlated with the transcriptional signature for T cells, but not B cells. The relationship between the cell proliferation response and T cell subsets was stronger for the CD8 T cell signature than for the CD4 T cell signature. To corroborate these findings, we undertook multiparameter flow cytometry of peripheral blood mononuclear cells (PBMCs) obtained in a subset of participants with contemporaneous PCR-positive infection and compared these to PBMCs from uninfected controls (Figure S5). Representation of the pooled multiparameter flow cytometry data by tSNE incorporating all T cells revealed new populations of CD4- and CD8-positive cells only in samples from infected participants, which also exhibited the highest levels of Ki67 staining as a marker of cell proliferation (Figure 3B), accompanied by HLA-DR expression as a marker of cell activation in exemplar cases (Figure 3C). In this subset of samples, only Ki67 staining of CD8 T cells was statistically enriched in infected individuals compared with controls (Figure 3D). Both CD4 and CD8 T cells showed statistically significant enrichment of Ki67 staining among selected memory T cell populations, but not naive T cells (Figure S5B). Of all other lymphocyte subsets, only NKT-like ($CD3^+CD56^+$) cells showed a significant increase in Ki67-positive staining (Figure S5C) but comprised on average 3% of circulating lymphocytes compared with T cells that comprised approximately 40% (Figure S5D).

Rapid clonal T cell expansion in response to SARS-CoV-2 infection associated with significant enrichment of SARS-CoV-2-reactive T cell receptors

To further evaluate the rapid T cell response to SARS-CoV-2 infection, we undertook sequencing of T cell receptor (TCR) α and β chains in longitudinal samples to reflect dynamic changes in the T cell clonal repertoire. The abundance of α and β chain

CellPress
OPEN ACCESS

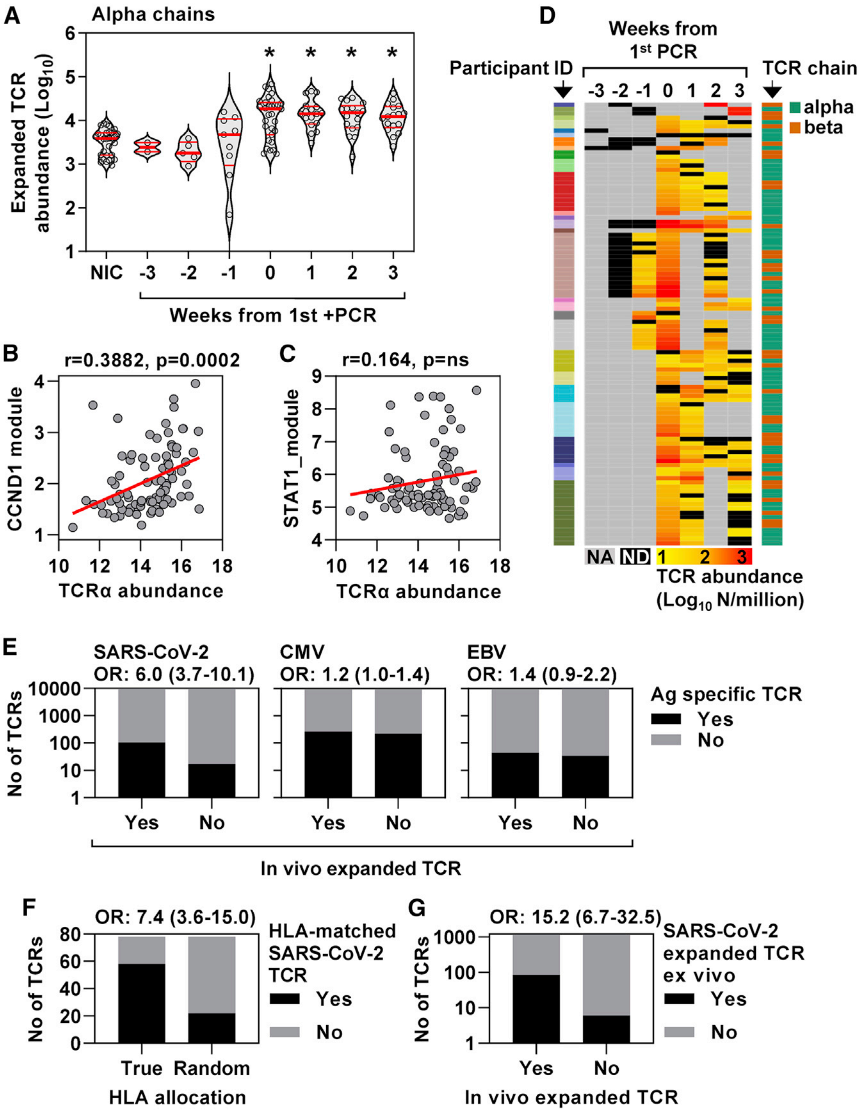

Figure 4. Cell proliferation response to co-incident SARS-CoV-2 infection associated with expansion of TCR clones enriched for SARS-CoV-2-reactive TCRs

(A) Enumeration of expanded TCR α chain abundance (per million total sequences) in non-infection controls and samples from infected individuals stratified by time from first positive PCR. Individual data points are shown with violin plots depicting median, IQR, and frequency distributions (*FDR < 0.05 by Kruskal-Wallis test for each group compared with NIC).

(B and C) Correlation of (B) CCND1 module and (C) STAT1 module with TCR α chain sequences ($\log_2$ per million sequences). Regression lines are shown in red, with R and p values for Spearman rank correlations.

(D) The dynamics of *in-vivo*-expanded TCRs (counts per million TCRs) identified as SARS-CoV-2 reactive in VDJdb, displayed as a heatmap in which each row is an individual TCR (α or β gene, right-hand key) from individual participants (left-hand key). NA, no sample available; ND, not detected in sample.

(E) Number of TCR sequences (α and β genes) annotated for SARS-CoV-2, cytomegalovirus (CMV), and Epstein-Barr virus (EBV) in VDJdb matching either expanded or unexpanded TCR sequences from individuals with SARS-CoV-2 infection, giving the odds ratio (OR±95% CI, Fisher's exact test) for enrichment of antigen-specific TCR sequences in each case.

(F) Number of SARS-CoV-2-specific TCRs in VDJdb for which the reported HLA restriction (HLA A1 or HLA A2) matches the HLA haplotype of the individual in which the expansion was observed. The number is compared with the expected number of HLA matches if HLA allocation was random, and these numbers are used to derive the OR (±95% CI, Fisher's exact test).

(G) Number of *ex vivo* SARS-CoV-2 peptide-reactive TCR sequences (α and β genes) among expanded and unexpanded TCR sequences from individuals with SARS-CoV-2 infection, giving the OR (±95% CI, Fisher's exact test) for enrichment of virus-specific TCR sequences.

sequences in each sample were tightly co-correlated (Figure S6A). An expanded clone will increase or decrease in frequency depending on the sampling time point before and after the peak response. Therefore, we identified expanded TCR sequences as being statistically enriched at one time point compared with at least one other time point and summed the total number of expanded sequences for these TCRs at each time point per million of total TCR sequences (Figures S6B and S6C). These were compared with expanded sequences identified in the same way among a subset of six uninfected controls in whom we undertook TCR sequencing in samples from 5 successive weeks. By comparison to the pooled data from controls, a significant increase in expanded TCRs was evident in infected individuals by the time of the first positive PCR test up to a maximum abundance of >6% of total TCR sequences and persisted for at least 3 weeks for both α and β chains (Figures 4A and S6). The abundance of expanded TCR sequences correlated significantly with the CCND1, but not the STAT1, regulated module, consistent with the hypothesis that the proliferation response reflected expansion of T cell clones (Figures 4B and 4C).

T cell clonal expansion was not explained by changes in MAIT cell- or NKT cell-associated TCR sequences (Figures S6E and S6F). A total of 102 expanded SARS-CoV-2-reactive α or β chain TCR sequences from the contemporary VDJdb catalog[12] were evident in 28 of the 41 individuals with incident PCR-positive infection (Figure 4D; Data S2). In all but one of these individuals where a contemporaneous sample was available, expanded SARS-CoV-2-reactive TCRs were present by the time of first positive PCR test (Figure 4D). Expanded α and β TCRs among infected individuals were significantly enriched for SARS-CoV-2-reactive TCRs in VDJdb (Figure 4E) with similar odds ratios (ORs) for enrichment of α and β chains (Figure S7A). In contrast, there was no enrichment of either CMV or Epstein-Barr virus

(EBV)-reactive TCRs, which would represent non-specific bystander memory T cell proliferation. We also found a significant association between the reported HLA restriction of the TCRs in the database and the HLA type of the individuals in whom the matched expanded TCR was observed (Figure 4F).

The analysis above is dependent on accurate annotation of public SARS-CoV-2-reactive TCR sequences and is skewed toward anti-spike CD8 responses, reflecting the preponderance of MHC multimer sorted T cells in VDJdb. Therefore, it is likely to underestimate the proportion of SARS-COV-2 expanded TCR sequences associated with incident infection in our participants because it will not include either private or $CD4^+$ TCR specificities. To address this limitation and further confirm the significant enrichment of SARS-CoV-2-reactive T cell clones within our TCR analysis, we compared expanded α and β TCR sequences *in vivo* with antigen-specific TCR sequences that expanded following *ex vivo* peptide stimulation in three individuals with convalescent PMBC samples available at 16 weeks after infection. Antigen-specific responses by CD4 and CD8 T cells to both structural and non-structural viral peptides in these samples were confirmed by flow cytometry (Figure S7B) and associated with stimulus-specific expansion of TCR sequences (Figure S7C). The *ex vivo* SARS-CoV-2 peptide-reactive TCRs were significantly enriched among *in-vivo*-expanded TCRs within the same individuals (Figure 4G). Likewise, the *in-vivo*-expanded TCR sequences were significantly enriched among *ex vivo* SARS-CoV-2 peptide-reactive TCRs (Figure S7D). As predicted above, the overlap between *in vivo* and *ex vivo* SARS-CoV-2-associated TCR responses showed a higher OR than the overlap of *in-vivo*-expanded TCRs with the SARS-CoV-2 TCR catalog in VDJdb.

Circulating virus-specific antibodies lag 2 weeks behind transient increase in immunoglobulin gene expression in response to SARS-CoV-2 infection

The finding that the CCND1-regulated module did not correlate with our B cell signature does not exclude a B cell response. Emergence of antibodies to SARS-CoV-2 has been reported as early as 5 days after symptom onset.[13] Flow cytometry of a subset of PBMCs available from those with contemporaneous PCR-positive infection showed no significant increase in frequency of selected B cell subsets, or among proliferating (Ki67-positive) B cells, compared with uninfected controls (Figures 5A and 5B). Interestingly, in blood transcriptional profiles we found increased expression of immunoglobulin (Ig) constant heavy- and light-chain transcripts, which peaked at the time of first PCR virus detection but was evident from 1 week before to 2 weeks after first PCR detection (Figures 5C and 5D). The increase in Ig gene expression in blood was less sustained than TCR expansion and returned to baseline by 3 weeks after the first positive PCR. In contrast, circulating antibodies to SARS-CoV-2 S1 spike protein that correlate with virus neutralization were not detectable until 1 week after the incident infection (Figure 5E) and continued to increase in this cohort for 8 weeks.[8,9]

DISCUSSION

To the best of our knowledge, we report the earliest *in vivo* immune responses to natural SARS-CoV-2 infection available to date, enabled by serial sampling of individuals at risk of infection during the peak of the first epidemic wave in London. The general paradigm for early antiviral host defense is dominated by induction of type 1 IFNs. Attenuated responses as a result of autoantibodies to type 1 IFNs and genetic polymorphisms associated with reduced expression of a type 1 IFN receptor subunit or with reduced expression of the IFN-inducible oligoadenylate synthetase gene cluster have all been associated with severe disease.[1–3] These provide strong evidence that type 1 IFN responses contribute to effective protection against severe SARS-CoV-2 infection. We show that type 1 IFN responses can precede PCR detection of the virus and therefore may exert their protective effects in the earliest phases of infection, independent of symptoms. We propose that such early detection of IFN-inducible genes in the blood transcriptome may arise from localized immune responses as a result of leukocyte trafficking through lymphoid tissues or the site of infection and may provide greater sensitivity than detection of circulating IFNs. As we have previously reported, an additional translational application of this finding is the detection of IFN-inducible transcripts in blood as a diagnostic biomarker of early viral infection that may precede PCR detection of the virus and symptoms.[14]

Alongside type 1 IFN responses, we detected an early cell proliferation response in the blood transcriptome, which we primarily attribute to CD8, and to a lesser extent CD4, T cell proliferation by correlation with cell-type-specific transcriptional modules, corroborated by flow cytometry to show a significant increase in Ki67-positive CD8 T cells and TCR sequencing to show expansion of T cell clones. While type 1 IFN responses were evident in a range of other acute respiratory virus infections modeled in human challenge experiments,[11] the early T cell response to SARS-CoV-2 in our study was significantly greater than in other viral infections. By comparison with current databases of SARS-CoV-2-specific and HLA A1/A2 matched TCRs in VDJdb and of TCRs that expand specifically in response to structural and non-structural virus peptide pools, we were able to show that *in-vivo*-expanded T cell clones were highly enriched for SARS-CoV-2-reactive cells and that these were already evident by the time of first positive virus PCR. In individuals with COVID-19, T cell reactivity has been reported as early as 5–10 days after the onset of symptoms.[15] Importantly, in one report, T cell proliferative responses to SARS-CoV-2 were evident in 92% of family contacts of COVID-19 cases independently of serostatus,[16] and some people may have pre-existing cross-reactive T cells arising from previous seasonal coronavirus exposure.[16–21] These may be expected to contribute to early viral clearance, as suggested in a subset of individuals with evidence of abortive SARS-CoV-2 infection[5] and analogous to findings in influenza.[22–24] We note that quantification of blood transcriptional perturbation revealed some outliers in the uninfected control group (Figure 1A). It is interesting to speculate whether these may also represent immune responses in an abortive infection.

If cross-reactivity were the primary driver of rapid T cell responses to SARS-CoV-2 infection, the fact that the early proliferative response discriminated infected and uninfected individuals with an AUROC of 0.92 would require pre-existing T cell priming to be a near ubiquitous feature of asymptomatic or non-severe

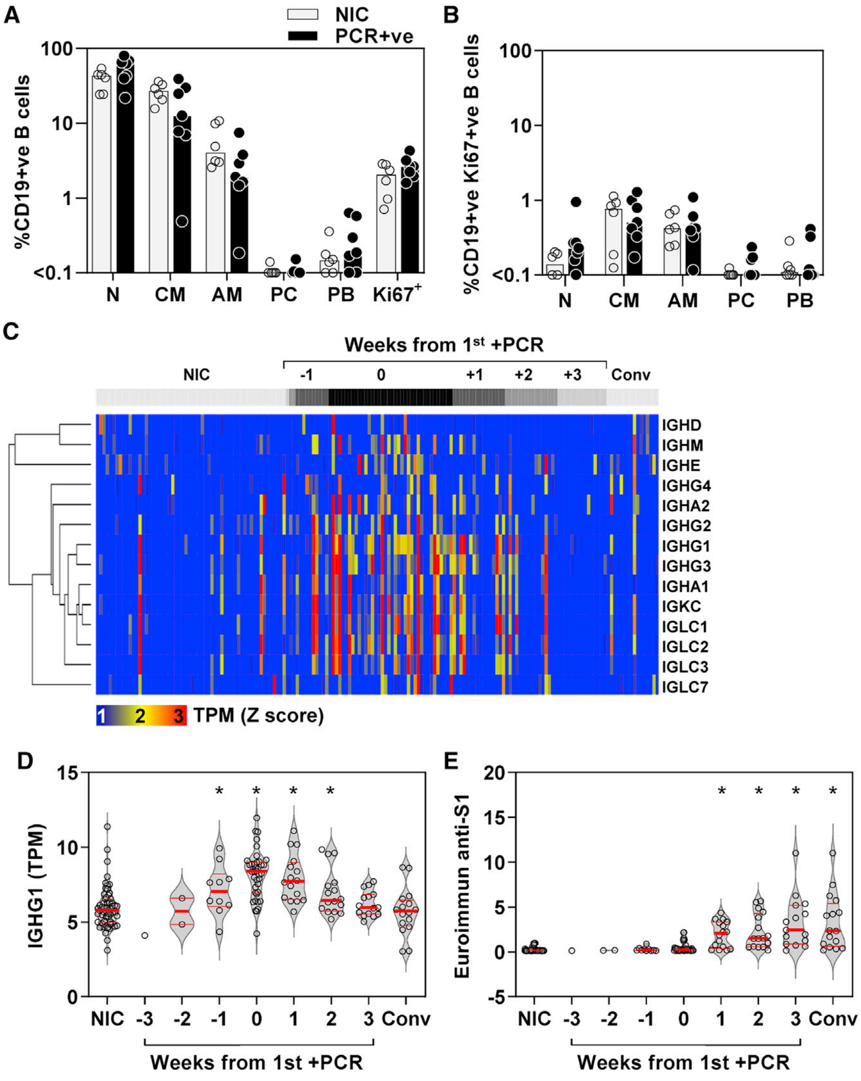

Figure 5. Enriched immunoglobulin gene expression and antibody response to incident SARS-CoV-2 infection

(A and B) (A) Frequency of B cell (N, naive; CM, classical memory; AM, activated memory; PC, plasma cells; PB, plasmablasts) subsets among total CD19-positive cells and (B) Ki67-positive CD19 cells in PBMCs from six NICs and seven individuals with co-incident infection, showing individual data points and the median (bars).

(C) Heatmap of immunoglobulin constant heavy- and light-chain gene expression in blood per individual (columns) stratified by time to first positive SARS-CoV-2 infection, compared with NIC and convalescent (Conv) samples 5–6 months after incident infection, presented as standardized (*Z*) scores of transcripts per million (TPM) using mean and SD of NIC.

(D and E) Blood TPM of (D) IGHG1 and (E) relative IgG anti-S1 antibody levels stratified by time to first positive SARS-CoV-2 infection, compared with NIC and Conv samples. Individual data points shown with violin plots depicting median, IQR, and frequency distributions (*FDR < 0.05 by Kruskal-Wallis test for each group compared with NIC).

infection. Consistent with this hypothesis, among the largest studies of pre-pandemic blood samples, heterologous T cell reactivity to SARS-CoV-2 peptides with proven similarity to those of pre-existing seasonal coronaviruses has been reported in 81%.[21] In this context, we hypothesize that the variation in T cell proliferative response and the lack of its correlation with type 1 IFN responses may be explained by differential levels of T cell priming in individual participants. We also identified a similarly rapid B cell response represented by transient enrichment of Ig gene expression in blood, but we were unable to corroborate any significant changes in the frequency of B cell subsets in a small sample available for flow cytometry. We speculate that the B cell response identified in the blood transcriptome may represent non-specific or sub-optimal activation of Ig gene expression, and because protective anti-S1 antibodies only became detectable after a 2-week lag, we hypothesize that the B cell response was unlikely to contribute significantly to rapid viral clearance in asymptomatic and non-severe infection.

Limitations of the study

Our study has some important limitations. The precise time of exposure to SARS-CoV-2 or transmission of infection was not possible to determine. This was offset by including longitudinal samples in 12 subjects obtained before detection of incident infection by PCR, providing enough statistical power to show that both type 1 IFN and cell proliferation responses were statistically enriched in the week before the first positive PCR result. We had limited access to PBMCs to assess frequency, phenotypic, and functional characteristics of SARS-CoV-2-reactive T cells, preventing further validation of a predominant virus-specific CD8 T cell response to early infection. The accumulating database of SARS-CoV-2-specific TCR sequences allowed us to relate clonal T cell expansion with HLA-class-1-restricted antigen specificity. This only accounted for a small fraction of expanded sequences and does not exclude proliferation of bystander T cells. However, the lack of significant enrichment of CMV- or EBV-specific TCRs among expanded clones, typically a major component of bystander responses to early viral infection,[25] and the lack of enrichment for IFN-γ activity or other signatures of T cell activation in the blood transcriptome argue against generalized bystander T cell activation. We also confirmed that early *in vivo* TCR expansion in infected individuals was enriched for SARS-CoV-2 TCR expansion in response to *ex vivo* stimulation with viral peptides, but future studies will be required to undertake this analysis at single-cell level to establish the relative contribution of CD4 and CD8 T cell clones. Our focus on the blood compartment means that we do not have direct

measurements of responses at the site of host-pathogen interactions. Analysis of bulk RNA samples for transcriptional profiling and TCR sequencing restricted our ability to evaluate transcriptional heterogeneity at the cellular level, further characterize expanded T cell clones, or undertake TCR analysis with paired α/β chains. Most importantly, since less than 5% of infections lead to hospitalization,[26] our study design precluded comparison of severe and non-severe outcomes that would require substantially greater sample size. Nonetheless, our data reflect immune responses in asymptomatic and non-severe infection, which incorporate correlates of effective host defense to natural infection in a SARS-CoV-2 naive population, providing further evidence for the importance of early type 1 IFN and T cell responses. Human challenge experiments that control for variation in time and dose of exposure will offer the best opportunities to acquire the granular detail of early immune responses. Larger-scale studies will be required to assess the frequency of SARS-CoV-2 T cell reactivity in naive populations and determine whether early type 1 IFN or T cell responses predict outcomes.

Although vaccine rollout is likely to be the primary immunological strategy to control the pandemic,[27] understanding the determinants of effective natural immunity will remain a critical objective to enable risk stratification and novel vaccine design as the virus evolves. In particular, identification of the antigenic determinants of the earliest T cell responses in asymptomatic SARS-CoV-2 infection is a priority to inform development of potential universal coronavirus vaccines.

CONSORTIA

The members of COVIDsortium Investigators are Hakam Abbass, Aderonke Abiodun, Mashael Alfarih, Zoe Alldis, Daniel M. Altmann, Mervyn Andiapen, Jessica Artico, Joao Augusto, Georgina L. Baca, Anish Bhuva, Alex Boulter, Ruth Bowles, Rosemary J. Boyton, Olivia Bracken, Timothy Brooks, Natalie Bullock, Gabriella Captur, Benny Chain, Nicola Champion, Carmen Chan, Jorge Couto de Sousa, Xose Couto-Parada, Marie-Teresa Cutino-Moguel, Rhodri H. Davies, Keenan Dieobi-Anene, Karen Feehan, Malcolm Finlay, Marianna Fontana, Nasim Forooghi, Joseph M. Gibbons, Derek Gilroy, Peter Griffiths, Rishi K. Gupta, Matt Hamblin, Lauren M. Hickling, Aroon D. Hingorani, Lee Howes, Ivie Itua, Victor Jardim, Melanie Jensen, Meleri Jones, George Joy, Vikas Kapil, Jonathan Lambourne, W.Y. Jason Lee, Mala K. Maini, Vineela Mandadapu, Charlotte Manisty, Aine McKnight, Katia Menacho Medina, Celina Mfuko, Oliver Mitchelmore, James C. Moon, Mahdad Noursadeghi, Ben O'Brien, Ben Ollivere, Corinna Pade, Susana Palma, Kush Patel, Ruth Parker, Brian Piniera, Alicja Rapala, Amy Richards, Mathew Robathan, Genine Sambile, Amanda Semper, Andreas Seraphim, Angelique Smit, Michelle Sugimoto, George D. Thornton, Thomas A. Treibel, Arthur Tucker, Ana Valdes, Jessry Veerapen, Mohit Vijayakumar, Timothy Warner, Sophie Welch, Dylan Williams, Theresa Wodehouse, Lucinda Wynne, and Dan Zahedi.

STAR★METHODS

Detailed methods are provided in the online version of this paper and include the following:

- KEY RESOURCES TABLE
- RESOURCE AVAILABILITY
  - Lead contact
  - Materials availability
  - Data and code availability
- EXPERIMENTAL MODEL AND SUBJECT DETAILS
  - Study design
  - Ethical approval
- METHOD DETAILS
  - Blood RNA sequencing
  - Peripheral blood mononuclear cells (PBMCs)
  - Flow cytometry
  - *Ex vivo* T cell stimulation
- QUANTIFICATION AND STATISTICAL ANALYSIS
  - Blood RNA sequencing data analysis
  - Transcriptional modules
  - Data from human challenge studies
  - Flow cytometry data analysis
  - T cell receptor sequencing and analysis

SUPPLEMENTAL INFORMATION

Supplemental information can be found online at https://doi.org/10.1016/j.xcrm.2022.100557.

ACKNOWLEDGMENTS

Funding for COVIDsortium was donated by individuals, charitable Trusts, and corporations, including Goldman Sachs, Kenneth C. Griffin, The Guy Foundation, GW Pharmaceuticals, Kusuma Trust, and Jagclif Charitable Trust, and enabled by Barts Charity with support from UCLH Charity. R.K.G. is funded by the National Institute for Health Research (DRF-2018-11-ST2-004). J.C.M., C.M., and T.A.T. are directly and indirectly supported by the University College London Hospitals NHS Foundation Trust (UCLH) and Barts NIHR Biomedical Research Centers and through the British Heart Foundation (BHF) Accelerator Award (AA/18/6/34223). T.A.T. is funded by a BHF Intermediate Research Fellowship (FS/19/35/34374). M.N. is supported by the Wellcome Trust (207511/Z/17/Z) and by NIHR Biomedical Research Funding to UCL and UCLH. M.K.M. is supported by UKRI/NIHR UK-CIC and Wellcome Trust (214191/Z/18/Z). G.S.T. is supported by a UK Medical Research Council Clinician Scientist Fellowship (MR/N007727/1). R.J.B. and D.M.A. are supported by UKRI/MRC Newton (MR/S019553/1, MR/R02622X/1, MR/V036939/1, MR/W020610/1), NIHR Imperial Biomedical Research Center (BRC): ITMAT, Cystic Fibrosis Trust SRC (2019SRC015), NIHR EME Fast Track (NIHR134607), NIHR Long Covid (COV-LT2-0027), Innovate Uk (SBRI 10008614), and Horizon 2020 Marie Skłodowska-Curie Innovative Training Network (ITN) European Training Network (no. 860325). A.M. is supported by Rosetrees Trust, The John Black Charitable Foundation, and Medical College of St Bartholomew's Hospital Trust. B.M.C. is supported by the Rosetrees Trust and the NIHR UCLH BRC.

AUTHOR CONTRIBUTIONS

Study conception, design, and oversight, G.C., C.M., T.A.T., J.C.M., and M.N.; data acquisition, A.C., J.R., G.N., L.S., A.R.B., C.P., J.M.G., and M.W.; data analysis and interpretation, A.C., J.R., L.S., G.P., R.K.G., A.R.B., A.W., T.R., M.R.d.M., B.S.-M.D.D.E., J.A.G.-A., A.S., T.B., D.M.A., R.J.B., A.M., G.S.T., M.K.M., B.M.C., and M.N.

DECLARATION OF INTERESTS

The authors declare no competing interests.

CellPress
OPEN ACCESS

Received: April 14, 2021
Revised: November 22, 2021
Accepted: February 9, 2022
Pubilshed: March 4, 2022

REFERENCES

1. Bastard, P., Rosen, L.B., Zhang, Q., Michailidis, E., Hoffmann, H.-H., Zhang, Y., Dorgham, K., Philippot, Q., Rosain, J., Béziat, V., et al. (2020). Autoantibodies against type I IFNs in patients with life-threatening COVID-19. Science *370*, eabd4585.

2. Pairo-Castineira, E., Clohisey, S., Klaric, L., Bretherick, A.D., Rawlik, K., Pasko, D., Walker, S., Parkinson, N., Fourman, M.H., Russell, C.D., et al. (2021). Genetic mechanisms of critical illness in COVID-19. Nature *591*, 92–98.

3. Zhang, Q., Bastard, P., Liu, Z., Le Pen, J., Moncada-Velez, M., Chen, J., Ogishi, M., Sabli, I.K.D., Hodeib, S., Korol, C., et al. (2020). Inborn errors of type I IFN immunity in patients with life-threatening COVID-19. Science *370*, eabd4570.

4. Boyton, R.J., and Altmann, D.M. (2021). The immunology of asymptomatic SARS-CoV-2 infection: what are the key questions? Nat. Rev. Immunol. *21*, 762–768.

5. Swadling, L., Diniz, M.O., Schmidt, N.M., Amin, O.E., Chandran, A., Shaw, E., Pade, C., Gibbons, J.M., Le Bert, N., Tan, A.T., et al. (2022). Pre-existing polymerase-specific T cells expand in abortive seronegative SARS-CoV-2. Nature *601*, 110–117.

6. Treibel, T.A., Manisty, C., Burton, M., McKnight, Á., Lambourne, J., Augusto, J.B., Couto-Parada, X., Cutino-Moguel, T., Noursadeghi, M., and Moon, J.C. (2020). COVID-19: PCR screening of asymptomatic health-care workers at London hospital. Lancet *395*, 1608–1610.

7. Treibel, T.A., Manisty, C., Andiapen, M., Pade, C., Jensen, M., Fontana, M., Couto-Parada, X., Cutino-Moguel, T., Noursadeghi, M., and Moon, J.C. (2020). Asymptomatic health-care worker screening during the COVID-19 pandemic - authors' reply. Lancet *396*, 1394–1395.

8. Manisty, C., Treibel, T.A., Jensen, M., Semper, A., Joy, G., Gupta, R.K., Cutino-Moguel, T., Andiapen, M., Jones, J., Taylor, S., et al. (2021). Time series analysis and mechanistic modelling of heterogeneity and sero-reversion in antibody responses to mild SARS-CoV-2 infection. EBioMedicine *65*, 103259.

9. Reynolds, C.J., Swadling, L., Gibbons, J.M., Pade, C., Jensen, M.P., Diniz, M.O., Schmidt, N.M., Butler, D.K., Amin, O.E., Bailey, S.N.L., et al. (2020). Discordant neutralizing antibody and T cell responses in asymptomatic and mild SARS-CoV-2 infection. Sci. Immunol. *5*, eabf3698.

10. Augusto, J.B., Menacho, K., Andiapen, M., Bowles, R., Burton, M., Welch, S., Bhuva, A.N., Seraphim, A., Pade, C., Joy, G., et al. (2020). Healthcare Workers Bioresource: study outline and baseline characteristics of a prospective healthcare worker cohort to study immune protection and pathogenesis in COVID-19. Wellcome Open Res. *5*, 179.

11. Liu, T.-Y., Burke, T., Park, L.P., Woods, C.W., Zaas, A.K., Ginsburg, G.S., and Hero, A.O. (2016). An individualized predictor of health and disease using paired reference and target samples. BMC Bioinformatics *17*, 47.

12. Bagaev, D.V., Vroomans, R.M.A., Samir, J., Stervbo, U., Rius, C., Dolton, G., Greenshields-Watson, A., Attaf, M., Egorov, E.S., Zvyagin, I.V., et al. (2020). VDJdb in 2019: database extension, new analysis infrastructure and a T-cell receptor motif compendium. Nucleic Acids Res. *48*, D1057–D1062.

13. Rydyznski Moderbacher, C., Ramirez, S.I., Dan, J.M., Grifoni, A., Hastie, K.M., Weiskopf, D., Belanger, S., Abbott, R.K., Kim, C., Choi, J., et al. (2020). Antigen-specific adaptive immunity to SARS-CoV-2 in acute COVID-19 and associations with age and disease severity. Cell *183*, 996–1012.e19.

14. Gupta, R.K., Rosenheim, J., Bell, L.C., Chandran, A., Guerra-Assuncao, J.A., Pollara, G., Whelan, M., Artico, J., Joy, G., Kurdi, H., et al. (2021). Blood transcriptional biomarkers of acute viral infection for detection of pre-symptomatic SARS-CoV-2 infection: a nested, case-control diagnostic accuracy study. Lancet Microbe *2*, e508–e517.

15. Tan, A.T., Linster, M., Tan, C.W., Le Bert, N., Chia, W.N., Kunasegaran, K., Zhuang, Y., Tham, C.Y.L., Chia, A., Smith, G.J.D., et al. (2021). Early induction of functional SARS-CoV-2-specific T cells associates with rapid viral clearance and mild disease in COVID-19 patients. Cell Rep *34*, 108728.

16. Sekine, T., Perez-Potti, A., Rivera-Ballesteros, O., Strålin, K., Gorin, J.-B., Olsson, A., Llewellyn-Lacey, S., Kamal, H., Bogdanovic, G., Muschiol, S., et al. (2020). Robust T cell immunity in convalescent individuals with asymptomatic or mild COVID-19. Cell *183*, 158–168.e14.

17. Le Bert, N., Tan, A.T., Kunasegaran, K., Tham, C.Y.L., Hafezi, M., Chia, A., Chng, M.H.Y., Lin, M., Tan, N., Linster, M., et al. (2020). SARS-CoV-2-specific T cell immunity in cases of COVID-19 and SARS, and uninfected controls. Nature *584*, 457–462.

18. Grifoni, A., Weiskopf, D., Ramirez, S.I., Mateus, J., Dan, J.M., Moderbacher, C.R., Rawlings, S.A., Sutherland, A., Premkumar, L., Jadi, R.S., et al. (2020). Targets of T Cell responses to SARS-CoV-2 coronavirus in humans with COVID-19 disease and unexposed individuals. Cell *181*, 1489–1501.e15.

19. Braun, J., Loyal, L., Frentsch, M., Wendisch, D., Georg, P., Kurth, F., Hippenstiel, S., Dingeldey, M., Kruse, B., Fauchere, F., et al. (2020). SARS-CoV-2-reactive T cells in healthy donors and patients with COVID-19. Nature *587*, 270–274.

20. Mateus, J., Grifoni, A., Tarke, A., Sidney, J., Ramirez, S.I., Dan, J.M., Burger, Z.C., Rawlings, S.A., Smith, D.M., Phillips, E., et al. (2020). Selective and cross-reactive SARS-CoV-2 T cell epitopes in unexposed humans. Science *370*, 89–94.

21. Nelde, A., Bilich, T., Heitmann, J.S., Maringer, Y., Salih, H.R., Roerden, M., Lübke, M., Bauer, J., Rieth, J., Wacker, M., et al. (2021). SARS-CoV-2-derived peptides define heterologous and COVID-19-induced T cell recognition. Nat. Immunol. *22*, 74–85.

22. Sridhar, S., Begom, S., Bermingham, A., Hoschler, K., Adamson, W., Carman, W., Bean, T., Barclay, W., Deeks, J.J., and Lalvani, A. (2013). Cellular immune correlates of protection against symptomatic pandemic influenza. Nat. Med. *19*, 1305–1312.

23. Wilkinson, T.M., Li, C.K.F., Chui, C.S.C., Huang, A.K.Y., Perkins, M., Liebner, J.C., Lambkin-Williams, R., Gilbert, A., Oxford, J., Nicholas, B., et al. (2012). Preexisting influenza-specific CD4 + T cells correlate with disease protection against influenza challenge in humans. Nat. Med. *18*, 274–280.

24. Hayward, A.C., Wang, L., Goonetilleke, N., Fragaszy, E.B., Bermingham, A., Copas, A., Dukes, O., Millett, E.R.C., Nazareth, I., Nguyen-Van-Tam, J.S., et al. (2015). Natural T cell-mediated protection against seasonal and pandemic influenza. Results of the flu watch cohort study. Am. J. Respir. Crit. Care Med. *191*, 1422–1431.

25. Sandalova, E., Laccabue, D., Boni, C., Tan, A.T., Fink, K., Ooi, E.E., Chua, R., Shafaeddin Schreve, B., Ferrari, C., and Bertoletti, A. (2010). Contribution of herpesvirus specific CD8 T cells to anti-viral T cell response in humans. Plos Pathog. *6*, e1001051.

26. Knock E, Whittles L, Lees J, Perez Guzman P, Verity R, Fitzjohn R, Gaythorpe K, Imai N, Hinsley W, Okell L, et al. Report 41: The 2020 SARS-CoV-2 epidemic in England: key epidemiological drivers and impact of interventions [Internet]. 2020 Dec [cited 2021 Mar 17]. Available from: http://spiral.imperial.ac.uk/handle/10044/1/85146

27. Dagan, N., Barda, N., Kepten, E., Miron, O., Perchik, S., Katz, M.A., Hernán, M.A., Lipsitch, M., Reis, B., and Balicer, R.D. (2021). BNT162b2 mRNA Covid-19 vaccine in a nationwide mass vaccination setting. N. Engl. J. Med. *384*, 1412–1423.

28. Roe, J., Venturini, C., Gupta, R.K., Gurry, C., Chain, B.M., Sun, Y., Southern, J., Jackson, C., Lipman, M.C., Miller, R.F., et al. (2020). Blood transcriptomic stratification of short-term risk in contacts of tuberculosis. Clin. Infect Dis. *70*, 731–737.

29. Bray, N.L., Pimentel, H., Melsted, P., and Pachter, L. (2016). Near-optimal probabilistic RNA-seq quantification. Nat. Biotechnol. *34*, 525–527.

30. Soneson, C., Love, M.I., and Robinson, M.D. (2016). Differential analyses for RNA-seq: transcript-level estimates improve gene-level inferences. F1000Research *4*, 1521.
31. Durinck, S., Moreau, Y., Kasprzyk, A., Davis, S., De Moor, B., Brazma, A., and Huber, W. (2005). BioMart and Bioconductor: a powerful link between biological databases and microarray data analysis. Bioinformatics *21*, 3439–3440.
32. Leek, J.T., Johnson, W.E., Parker, H.S., Jaffe, A.E., and Storey, J.D. (2012). The sva package for removing batch effects and other unwanted variation in high-throughput experiments. Bioinformatics *28*, 882–883.
33. Gonçalves, A.N.A., Lever, M., Russo, P.S.T., Gomes-Correia, B., Urbanski, A.H., Pollara, G., Noursadeghi, M., Maracaja-Coutinho, V., and Nakaya, H.I. (2019). Assessing the impact of sample heterogeneity on transcriptome analysis of human diseases using MDP webtool. Front Genet. *10*, 971.
34. Jacomy, M., Venturini, T., Heymann, S., and Bastian, M. (2014). ForceAtlas2, a continuous graph layout algorithm for handy network visualization designed for the Gephi software. Muldoon MR. PLoS ONE *9*, e98679.
35. Fang, H., Knezevic, B., Burnham, K.L., and Knight, J.C. (2016). XGR software for enhanced interpretation of genomic summary data, illustrated by application to immunological traits. Genome Med. *8*, 129.
36. Turner, C.T., Brown, J., Shaw, E., Uddin, I., Tsaliki, E., Roe, J.K., Pollara, G., Sun, Y., Heather, J.M., Lipman, M., et al. (2021). Persistent T cell repertoire perturbation and T cell activation in HIV after Long term treatment. Front Immunol. *12*, 634489.
37. Weight, C.M., Venturini, C., Pojar, S., Jochems, S.P., Reiné, J., Nikolaou, E., Solórzano, C., Noursadeghi, M., Brown, J.S., Ferreira, D.M., et al. (2019). Microinvasion by Streptococcus pneumoniae induces epithelial innate immunity during colonisation at the human mucosal surface. Nat. Commun. *10*, 3060.
38. Pollara, G., Turner, C.T., Rosenheim, J., Chandran, A., Bell, L.C.K., Khan, A., Patel, A., Peralta, L.F., Folino, A., Akarca, A., et al. (2021). Exaggerated IL-17A activity in human *in vivo* recall responses discriminates active tuberculosis from latent infection and cured disease. Sci. Transl Med. *13*, eabg7673.
39. Pollara, G., Murray, M.J., Heather, J.M., Byng-Maddick, R., Guppy, N., Ellis, M., Turner, C.T., Chain, B.M., and Noursadeghi, M. (2017). Validation of immune cell modules in multicellular transcriptomic data. PLoS One *12*, e0169271.
40. Oakes, T., Heather, J.M., Best, K., Byng-Maddick, R., Husovsky, C., Ismail, M., Joshi, K., Maxwell, G., Noursadeghi, M., Riddell, N., et al. (2017). Quantitative Characterization of the T cell receptor repertoire of naïve and memory subsets using an integrated experimental and Computational pipeline which is robust, economical, and versatile. Front Immunol. *8*, 1267.
41. Uddin, I., Joshi, K., Oakes, T., Heather, J.M., Swanton, C., and TRACERx consortium; and Chain, B. (2019). An economical, quantitative, and robust protocol for high-throughput T cell receptor sequencing from tumor or blood. Methods Mol. Biol. *1884*, 15–42.
42. Peacock, T., Heather, J.M., Ronel, T., and Chain, B. (2021). Decombinator V4: an improved AIRR compliant-software package for T-cell receptor sequence annotation? Bioinformatics *37*, 876–878.

STAR★METHODS

KEY RESOURCES TABLE

REAGENT or RESOURCE	SOURCE	IDENTIFIER
Antibodies		
BV421-conjugated anti 4-1BB, clone 4B4-1	Biolegend	Cat no: 309820; RRID:AB_2563830
PE-conjugated anti Blimp-1, clone 6D3	BD biosciences	Cat no: 564702; RRID:AB_2738901
FITC-conjugated anti CCR7, clone 150503	BD biosciences	Cat no: 561271; RRID:AB_10561679
BV711-conjugated anti CD11c, clone 3.9	Biolegend	Cat no: 301630; RRID:AB_2562192
APC-conjugated anti CD138, clone DL-101	Biolegend	Cat no: 352308; RRID:AB_10896946
V500-conjugated anti CD14, clone M5E2	BD biosciences	Cat no: 561391; RRID:AB_10611856
Pe-conjugated anti CD161, clone 191B8	Miltenyi	Cat no: 130-092-677; RRID:AB_871632
BV786-conjugated anti CD19, clone SJ25C1	BD biosciences	Cat no: 740968; RRID:AB_2740593
AF700-conjugated anti CD20, clone 2H7	BD biosciences	Cat no: 560631; RRID:AB_1727447
BV421-conjugated anti CD21, clone B-Ly4	BD biosciences	Cat no: 562966; RRID:AB_2737921
PE/Cy7-conjugated anti CD24, clone ML5	Biolegend	Cat no: 311120; RRID:AB_2259843
BUV395-conjugated anti CD27, clone L128	BD biosciences	Cat no: 563815; RRID:AB_2744349
BUV805-conjugated anti CD3, clone UCHT1	BD biosciences	Cat no: 612895; RRID:AB_2870183
BV510-conjugated anti CD3, clone OKT3	Biolegend	Cat no: 317332; RRID:AB_2561943
PE-dazzle-conjugated anti CD38, clone HIT2	Biolegend	Cat no: 303538; RRID:AB_2564105
BUV395-conjugated anti CD4, clone SK3	BD biosciences	Cat no: 563550; RRID:AB_2738273
BV711-conjugated anti CD45RA, clone HI100	BD biosciences	Cat no: 563733; RRID:AB_2738392
Pe-Dazzle594-conjugated anti CD56, clone QA17A16	Biolegend	Cat no: 392410; RRID:AB_2728406
BV605-conjugated anti CD69, clone FN50	Biolegend	Cat no: 310938; RRID:AB_2562307
APC-Cy7-conjugated anti CD71, clone CY1G4	Biolegend	Cat no: 334110; RRID:AB_2563117
PerCP/Cy5.5-conjugated anti CD71, clone Cy1G4	Biolegend	Cat no: 334114; RRID:AB_2563175
AlexaFluor700-conjugated anti CD8a, clone RPA-T8	Biolegend	Cat no: 301028; RRID:AB_493745
BV605-conjugated anti CXCR4, clone I2G5	Biolegend	Cat no: 306521; RRID:AB_2562443
V500-conjugated anti HLA-DR, clone G46-6	BD biosciences	Cat no: 561224; RRID:AB_10563765
APC-conjugated anti IFNg, clone 4S.B3	Biolegend	Cat no: 502512; RRID:AB_315237
BUV805-conjugated anti IgD, clone IA6-2	BD biosciences	Cat no: 742039; RRID:AB_2871332
APC/Cy7-conjugated anti IgM, clone MHM-88	Biolegend	Cat no: 314520; RRID:AB_10900422
Pe-Cy7-conjugated anti Ki67, clone 20Raj1	ThermoFisher	Cat no: 25-5699-42; RRID:AB_2573462
AF488-conjugated anti Ki67, clone B56	BD biosciences	Cat no: 561165; RRID:AB_10611866
AlexFluor647-conjugated anti OX40, clone Ber-ACT35	Biolegend	Cat no: 350018; RRID:AB_2571938
BB700-conjugated anti PD-1, clone EH12.1	BD biosciences	Cat no: 566460; RRID:AB_2744348
BV785-conjugated anti TCR Va7.2, clone 3C10	Biolegend	Cat no: 351722; RRID:AB_2566042
Funtional grade anti-CD28, clone CD28.2	ThermoFisher	Cat no; 16-0289-81; RRID:AB_468926

(*Continued on next page*)

Continued		
REAGENT or RESOURCE	SOURCE	IDENTIFIER
Biological samples		
Blood RNA samples	This study	N/A
Peripheral blood mononuclear cell samples	This study	N/A
Bacillus Calmette Guerin (BCG)	NIBSC (UK)	Cat no: 07/274
Chemicals, peptides, and recombinant proteins		
Recombinant human IL-2	Peprotech	Cat no: 200–02
SARS-CoV-2 overlapping peptide pools covering structural spike, nucleoprotein, membrane protein, NSP7, NSP12, NSP13	GL Biochem Shanghai	Custom
RLT buffer	Qiagen	Cat no: 79216
Ficoll-Hypaque Plus	GE healthcare	Cat no: 17-1440-03
Dimethyl sulfoxide solution	Sigma Aldrich	Cat no: 67-68-5
Fetal bovine serum	Sigma Aldrich	Cat no: F7524
Phosphate buffered saline	ThermoFisher	Cat no: 10010023
Brilliant violet buffer	BD biosciences	Cat no: 563794
Critical commercial assays		
Tempus^{TM} Blood RNA tubes	ThermoFisher	Cat no: 4342792
Tempus Spin RNA Isolation Kit	ThermoFisher	Cat no: 4380204
GlobinClear kit	ThermoFisher	Cat no: AM1980
TURBO DNA-free kit	ThermoFisher	Cat no: AM2238
Kappa Hyperprep kit	Roche	Cat no: 07962363001
Nextseq 500/550 High Output 75 cycle kit	Illumina	Cat no: 20024906
LIVE/DEAD^{TM} Fixable Blue Dead Cell Stain Kit	ThermoFisher	Cat no: L34962
Foxp3 / Transcription Factor staining buffer	eBioscience	Cat no: 00-5523-00
RNEasy kit	Qiagen	Cat no: 74004
Deposited data		
Blood RNAseq data from the present study cohort	ArrayExpress	ArrayExpress: E-MTAB-10022
Microarray transcriptional profiles from PPD stimulated PBMC	ArrayExpress	ArrayExpress: E-MTAB-11345
TCRseq data	NCBI Short Read Archive	NCBI Short Read Archive: SUB9362448
Supplemental Data S1 and Data S2	Mendeley Data	https://data.mendeley.com/datasets/vm4dvpxxdy/2 https://doi.org/10.17632/vm4dvpxxdy.2
Software and algorithms		
Kallisto	Bray et al., 2016	https://github.com/pachterlab/kallisto
Tximport	Bioconductor	https://bioconductor.org/packages/release/bioc/html/tximport.html
BioMart	Bioconductor	https://bioconductor.org/packages/release/bioc/html/biomaRt.html
sva package	Bioconductor	https://bioconductor.org/packages/release/bioc/html/sva.html
Ingenuity Pathway Analysis	Qiagen	https://digitalinsights.qiagen.com/products-overview/discovery-insights-portfolio/analysis-and-visualization/qiagen-ipa/
Gephi v0.9.2	Jacomy et al., 2014	https://gephi.org/
XGR	Fang et al., 2016	https://xgr.r-forge.r-project.org/

(Continued on next page)

CellPress
OPEN ACCESS

Continued

REAGENT or RESOURCE	SOURCE	IDENTIFIER
FlowJo v10.7.1	BD biosciences	N/A
Decombinator v4	Peacock et al., 2021	https://github.com/innate2adaptive/Decombinator
Other		
VDJdb database (accessed 1st November 2021)	Bagaev et al., 2020	https://vdjdb.cdr3.net/
Blood transcriptional profiles from selected human respiratory virus challenge studies	Gene Expression Omnibus	https://www.ncbi.nlm.nih.gov/geo/ (accession no: GSE73072)

RESOURCE AVAILABILITY

Lead contact

Further information and requests for resources and reagents should be directed to and will be fulfilled by the lead contact, Mahdad Noursadeghi (m.noursadeghi@ucl.ac.uk).

Materials availability

Requests for access to samples will be considered (subject to a material transfer agreement) by an access committee via an online application (https://covid-consortium.com/application-for-samples/), on the basis of availability and scientific merit within the scope of research ethics approvals, participant consent, and data governance. Responses to applications will be made within 4 weeks.

Data and code availability

- Open access to new transcriptional profiling data and essential anonymised metadata is available online through ArrayExpress: E-MTAB-10022 and ArrayExpress: E-MTAB-11345. TCR sequencing data are available at NCBI Short Read Archive: SUB9362448. Requests for access to individual de-identified participant level data will be considered (subject to a data transfer agreement) by an access committee via an online application (https://covid-consortium.com/application-for-samples/), on the basis of availability and scientific merit within the scope of research ethics approvals, participant consent, and data governance. Responses to applications will be made within 4 weeks.
- No custom code was generated for the present analysis.
- Any additional information required to reanalyze the data reported in this work paper is available from the Lead contact upon request.

EXPERIMENTAL MODEL AND SUBJECT DETAILS

Study design

We undertook a case control study nested within our COVIDsortium health care worker cohort. Participant screening, study design, sample collection, and sample processing have been described in detail previously.[6,7,9,10] Healthcare workers were recruited at St Bartholomew's Hospital, London, UK in the week of lockdown in the United Kingdom (between 23rd and 31st March 2020). Participants underwent weekly evaluation using a questionnaire and biological sample collection (including serological assays) for up to 16 weeks when fit to attend work at each visit, with further follow up samples collected at 6 months. Participants with available blood RNA samples who had PCR-confirmed SARS-CoV-2 infection (Roche cobas diagnostic test platform) at any time point were included as 'cases'. A subset of consecutively recruited participants without evidence of SARS-CoV-2 infection on nasopharyngeal swabs and who remained seronegative by both Euroimmun antiS1 spike protein and Roche anti-nucleocapsid protein throughout follow-up were included as uninfected controls. The baseline characteristics of the study participants are provided in Table S1.

Ethical approval

The study was approved by a UK Research Ethics Committee (South Central - Oxford A Research Ethics Committee, ref. 20/SC/0149). All participants provided written informed consent.

METHOD DETAILS

Blood RNA sequencing

Blood samples for RNA sequencing were collected in Tempus Blood RNA tubes. For 'cases', we included all available RNA samples, including convalescent samples at week 24 of follow-up for a subset of participants. For uninfected controls, we included baseline

samples only. Genome wide mRNA sequencing was performed as previously described.[28] Total blood RNA was extracted using the Tempus Spin RNA Isolation Kit (ThermoFisher). Globin mRNA and genomic DNA were removed using the GlobinClear kit (ThermoFisher) and the TURBO DNA-free kit (ThermoFisher) respectively. Transcriptional profiling of blood RNA samples was performed by RNA sequencing. cDNA libraries were generated using the Kappa Hyperprep kit (Roche), and sequencing was performed on the Illumina Nextseq using the Nextseq 500/550 High Output 75 cycle kit (Illumina) according to manufacturers' instructions, resulting in a median of 26 million (range, 19·8–32 · 4 million) 41 bp paired-end reads per sample. RNAseq data were mapped to the reference transcriptome (Ensembl Human GRCh38 release 100) using Kallisto.[29] The transcript-level output counts and transcripts per million (TPM) values were summed on gene level and annotated with Ensembl gene ID, gene name, and gene biotype using the R/Bioconductor packages tximport and BioMart.[30,31]

Peripheral blood mononuclear cells (PBMCs)

PBMC were isolated from heparinised blood by density centrifugation using Ficoll-Hypaque Plus (GE Healthcare). PBMC were frozen in 10% DMSO (Sigma-Aldrich) in Isopropanol containers (−1 °C/min) at 5 x10^6 PBMC/mL in cryovials. Thawing was performed by gentle agitation at 37 °C with rapid dilution in RPMI containing 10% fetal bovine serum (FBS; Sigma-Aldrich).

Flow cytometry

For multiparametric flow cytometry cells were plated in 96-well round-bottomed plates (0.five to one x10^6 per sample) and washed once in PBS (PBS; ThermoFisher) then stained with Blue fixable live/dead dye (ThermoFisher) for 20 min at 4 °C in PBS, followed by selected antibodies (see key resources table and Table S2). Cells were washed again in PBS and incubated with saturating concentrations of monoclonal antibodies against markers to be stained on the cell surface, diluted in 50% Brilliant violet buffer (BD biosciences) and 50% PBS for 30 min at 4 °C unless stated. After surface Ab staining cells were resuspended in fix/perm buffer (eBiosciences, Foxp3/Transcription Factor staining buffer kit, fix perm concentrate diluted 1:3 in fix/perm diluent) for 45-60 min at 4 °C. Cells were then washed in 1x perm buffer (10x perm buffer Foxp3/Transcription Factor staining buffer kit diluted to 1X in ddH2O) and saturating concentrations of intranuclear targets (Ki67) were stained in 1X perm buffer for 30-45 min 4 °C. Cells were washed twice in PBS then analyzed by flow cytometry using the LSR II flow cytometer (BD biosciences).

Ex vivo T cell stimulation

5x10^5 PBMC were cultured with anti-CD28 (0.5 μg/mL) and rhIL-2 (20 IU/mL) ±pools of overlapping peptides (2 ug/mL per peptide) covering structural proteins (spike, nucleoprotein and membrane protein) and replication transcription complex (RTC) proteins (NSP7, NSP12, NSP13), as detailed previously,[5,9] comprising pools of 15-mer peptides overlapping by 10 amino acids >80% purity (GL Biochem Shanghai). Intracellular IFNγ cytokine staining post-expansion was performed as detailed previously,[5] with brefeldin A being added on day 7 and PBMC harvested and stained 16-18 h later. In parallel, for T cell receptor sequencing, PBMC were collected on day 8, washed twice in sterile PBS, frozen at −80 °C in RLT buffer (Qiagen), before thawing and extracting RNA using the RNEasy kit (Qiagen) according to manufacturer's instructions.

QUANTIFICATION AND STATISTICAL ANALYSIS

Blood RNA sequencing data analysis

Sample processing batch effects were evaluated by principal component analysis at genome wide level (Figure S2A) and among the intersect of the 10% genes with least variable expression in each sample processing batch (Figure S2B). A batch effect evident in the least variant gene expression analysis was corrected using the ComBat function in the sva package in R, allocating samples with PC2 score <0 and >0 (in Figure S2B) to separate batches.[32] Principal component analysis (PCA) of the least low variance gene expression after batch correction showed no further separation of samples by processing batch (Figure S2C). Molecular degree of perturbation (MDP) was calculated as previously described.[33] Transcripts were included if more than one sample had a TPM count above the limit of detection, and the SD (SD) of TPM among uninfected controls was>0.5. The TPM values for each individual dataset were then transformed to a Z score using the mean and SD for each transcript among uninfected controls used as a standard reference. The MDP of each sample/dataset was then represented as the sum of all Z scores>2 divided by the total number of transcripts. Differential gene expression between datasets from individuals with co-incident infection and non-infection controls was identified using a Mann-Whitney test with false discovery rate <0.05 and absolute fold difference >1.5 (or Log_2 0.585). Analysis of upstream transcriptional regulation of the differentially expressed genes was performed using Ingenuity Pathway Analysis (Qiagen, Venlo, The Netherlands) and visualised as network diagram using the Force Atlas two algorithm in Gephi v0.9.2.[34] We depicted all statistically over-represented molecules (false discovery rate <0.05), predicted to be upstream of >2 target genes, and annotated with one of the following molecular functions: cytokine, transmembrane receptor, kinase and transcriptional regulator, representing the canonical components of molecular pathways responsible for transcriptional reprogramming in immune responses. The biological pathways represented by the upstream regulators were identified by Reactome pathway enrichment analysis using XGR[35] as previously described.[36,37] For visualization, 20 pathway groups were identified by hierarchical clustering of Jaccard indices to quantify similarity between the gene compositions of each pathway. For each group, the pathway with the largest total number of genes was then selected to provide a representative annotation.

CellPress
OPEN ACCESS

Transcriptional modules

To identify co-regulated gene networks used as transcriptional modules, we calculated the average correlation coefficient for pairwise correlations of the expression levels of each group of target genes associated with predicted upstream regulators in our transcriptomic dataset, and compared this to the distribution of average correlation coefficients obtained from random selection of equivalent sized groups of genes repeated 100 times. Groups of target genes with average correlation coefficients that exceeded the mean of the distribution of equivalent sized randomly selected groups by $\geq$ 2 SD (*Z* score $\geq$2) with false discovery rate <0.05 were identified as transcriptional modules representing the functional activity of the associated upstream regulator (Figure S2E). Independently derived Type 1 and Type 2 interferon inducible modules and cell-type specific transcriptional modules were described previously.[36,38,39] To derive an independent cell proliferation module, PBMC were isolated from BCG-vaccinated individuals and stimulated *in vitro* $2x10^5$ colony forming units of Bacillus Calmette–Guérin Russia (NIBSC) for 6 days to drive proliferation of antigen specific T cells. Stimulated and unstimulated PBMC were subjected to transcriptional profiling, differential gene expression and Reactome pathway enrichment analysis as previously described.[38] Differentially enriched transcripts annotated to the "Cell Cycle" Reactome pathway (Table S3) were used to derive a transcriptional signature for T cell proliferation. The expression of each module was represented by the geometric the mean log_2 TPM value of its constituent genes.

Data from human challenge studies

Publicly available data from previously published human viral challenge studies were downloaded from Gene Expression Omnibus (accession GSE73072). We calculated module scores for the STAT1 and CCND1 modules as the mean expression across all constituent genes, using log_2-transformed microarray data. Only participants who developed evidence of infection following inoculation were included, as per the original study definitions.[11] The peak enrichment of STAT1 and CCND1-regulated modules for each infected individual was calculated was represented by the highest log_2 TPM ratio to the mean of uninfected controls, across the time course of each dataset (Figure S4).

Flow cytometry data analysis

Flow cytometry data were analyzed using FlowJo (version 10.7.1 for mac, Tree Star). Single stain controls were prepared with cells or anti-mouse IgG beads (BD biosciences). Fluorescence minus one (FMOs) were used for gating. Selected cell populations were quantified using established combinations of antibody stains (Table S2). For tSNE an equal number (2223 cells) of CD4 and CD8 T cells from each of the seven control and nine PCR + samples were concatenated and tSNE was calculated on single cells expression values for the following markers: CD4, CD8, HLA-DR, Ki67, CD45RA, CCR7 (Iterations 1000, perplexity 30, eta 4979; KNN algorithm, Exact. Gradient algorithm, Barnes-Hut).

T cell receptor sequencing and analysis

The alpha and beta genes of the TCR were sequenced from all time points for which RNA was available within the first 4 weeks of the study for all participants who were PCR + at any time point, and for six randomly selected individuals who remained PCR- and seronegative throughout the study. The pipeline introduces unique molecular identifiers attached to individual cDNA molecules which allows correction for sequencing error PCR bias, and provides a quantitative and reproducible method of library preparation. Full details for both the experimental TCRseq library preparation and the subsequent TCR annotation (V, J and CDR3 annotation) using Decombinator V4 have been described previously.[40,41,42] Expanded TCRs were defined as any TCR which changed significantly between any two time points (Figure S6B-S6C). The boundaries (shown as blue dotted lines) were defined as the maximum TCR abundance which might be observed at time 2, given its abundance at time 1, assuming Poisson distribution of counts with $p < 0.0001$, to give a false discovery rate of <1 in 1000. TCR abundances are normalised for total number of TCRs sequenced in each sample, and expressed as counts/million. MAIT TCRs were defined as any TCR alpha containing TRAV1-2 paired with TRAJ12, TRA20 or TRAJ33. iNKT TCRs were defined as TCRs containing TRAV10 paired with TRAJ18. The VDJdb database[12] (https://vdjdb.cdr3.net/), accessed on 1st November 2021, was searched for any TCR annotated for CMV, EBV or SARS-Cov-2. TCRs annotated for multiple antigens were excluded.

The number of *in vivo* expanded TCRs defined as described above which matched the antigen annotated TCRs for each set of antigen was then calculated. As a control, we calculated the average overlap observed using 10 same size random sets of TCRs from the same individuals, instead of the expanded TCRs. The VDJdb included the HLA restriction of each TCR determined experimentally. We measured the association between the VDJdb HLA of each matched TCR, with the known HLA haplotype of the individual in which the expansion was observed. We considered a match "correct" if the individual had at least one allele which matched that of the annotated matched TCR. The statistical association was calculated by comparing the number of "correct" and "incorrect" HLA matches with the number expected if HLA of all individuals was randomly shuffled.

The *in vitro* peptide stimulated T cells from three individuals were TCR sequenced after *in vitro* expansion, and compared to cultures from the same pool of T cells cultured without antigen but under identical culture conditions. T cell receptors were considered expanded if the frequency of the TCR was at least eight-fold higher in the antigen stimulated than the control cultures.

Cell Reports Medicine, Volume *3*

Supplemental information

Rapid synchronous type 1 IFN

and virus-specific T cell responses characterize

first wave non-severe SARS-CoV-2 infections

Aneesh Chandran, Joshua Rosenheim, Gayathri Nageswaran, Leo Swadling, Gabriele Pollara, Rishi K. Gupta, Alice R. Burton, José Afonso Guerra-Assunção, Annemarie Woolston, Tahel Ronel, Corinna Pade, Joseph M. Gibbons, Blanca Sanz-Magallon Duque De Estrada, Marc Robert de Massy, Matthew Whelan, Amanda Semper, Tim Brooks, Daniel M. Altmann, Rosemary J. Boyton, Áine McKnight, Gabriella Captur, Charlotte Manisty, Thomas Alexander Treibel, James C. Moon, Gillian S. Tomlinson, Mala K. Maini, Benjamin M. Chain, Mahdad Noursadeghi, and COVIDsortium Investigators

Supplementary materials

Contents

Supplementary Tables 2

Table S1. Baseline characteristics of the study cohort, related to Figure 1 and STAR methods. 2

Table S2. Flow cytometry detection of lymphocyte subsets, related to Figure 3, Figure 5 and flow cytometry analysis in STAR methods. 3

Table S3: Genes comprising the T cell proliferation module derived from PPD stimulated PBMC, related to derivation of a T cell proliferation in module in STAR methods and Figure S5 4

Supplementary Figures 5

Figure S1. Study consort diagram, related to Figure 1 and to sample selection in case-control study design in STAR methods. 5

Figure S2. Batch correction, molecular degree of perturbation and identification of co-regulated modules in blood transcriptomic data, related to analysis of Blood RNA sequencing data in STAR methods, and Figure 1. 6

Figure S3. Bioinformatic analysis of modules derived from upstream regulator analysis of differentially expressed genes in blood transcriptome associated with SARS-CoV-2 infection, related to Figure 1, Figure 2 and Blood RNA sequencing analysis in STAR methods. 7

Figure S4. STAT1 and CCND1 regulated module expression over time in blood transcriptomic data from individuals experimentally infected with acute respiratory viruses, related to Figure 2. 8

Figure S5. Multiparametric flow cytometry of PBMC and frequency of selected lymphocytes and their activation/proliferative state, related to Figure 3. 9

Figure S6. Identification of expanded TCRs, expansion of TCR beta chain sequences and abundance of MAIT/iNKT cell sequences in SARS-CoV-2 infection, related to Figure 4 and analysis of blood TCR sequencing data in STAR methods. 10

Figure S7. Ex vivo T cell responses to structural and non-structural peptides 16 weeks post-infection, related to Figure 4 and analysis of blood TCR sequencing data in STAR methods. 11

Supplementary Tables

Table S1. Baseline characteristics of the study cohort, related to Figure 1 and STAR methods.

**Characteristic**	**Overall, N = 96**	**Cases, N = 41[1]**	**Controls, N = 55[1]**
Age	36 (27, 47)	36 (28, 46)	36 (26, 50)
Gender (self reported)			
Female	69 (72%)	28 (68%)	41 (75%)
Male	27 (28%)	13 (32%)	14 (25%)
Ethnicity			
White	66 (69%)	25 (62%)	41 (75%)
Black	6 (6·3%)	5 (12%)	1 (1·8%)
Asian	18 (19%)	9 (22%)	9 (16%)
Other	5 (5·3%)	1 (2·5%)	4 (7·3%)
Unknown	1	1	0
Samples			
1	67 (70%)	12 (29%)	55 (100%)
2	7 (7·3%)	7 (17%)	0 (0%)
3	6 (6·2%)	6 (15%)	0 (0%)
4	10 (10%)	10 (24%)	0 (0%)
5	6 (6·2%)	6 (15%)	0 (0%)
Case-defining symptoms	36 (38%)	31 (76%)	5 (9·1%)

[1]Statistics presented: median (IQR); n (%)

Table S2. Flow cytometry detection of lymphocyte subsets, related to Figure 3, Figure 5 and flow cytometry analysis in STAR methods.

**Cell types**		**Flow cytometry staining characteristics**
T cells	CD4	CD3+, CD56-, CD4+
	CD8	CD3+, CD56-, CD8+
	Naïve	CD3+, CD45RA+, CCR7+
	TCM	CD3+, CD45RA-, CCR7+
	TEM	CD3+, CD45RA-, CCR7-
	TEMRA	CD3+, CD45RA+, CCR7-
NK cells		CD3-, CD56+
NK T cells		CD3+, CD56+
MAIT cells		CD161++, TCR Va7.2+
B cells	Naïve B cells:	CD20+, CD19+, IgD+, CD27-, CD21+ CD24+ CD38+
	Classical memory:	CD20+, CD19+, IgD-, CD27+, CD21+, (CD38+/lo, CD24+)
	Activated memory:	CD20+, CD19+, IgD-, CD27+, CD21-, (CD38-, CD24-)
	Plasma cells:	CD20-, CD19+, IgD-, CD27++, CD38++, CD138+, (CD24-,)
	Plasmablasts:	CD20-, CD19+, CD38++, CD27 +, (CD138-; CD24-)

Table S3: Genes comprising the T cell proliferation module derived from PPD stimulated PBMC, related to derivation of a T cell proliferation in module in STAR methods and Figure S5

Gene symbol	Ensembl gene ID
AURKA	ENSG00000087586
BUB1	ENSG00000169679
BUB1B	ENSG00000156970
CCNA2	ENSG00000145386
CCNB1	ENSG00000134057
CCNB2	ENSG00000157456
CDCA5	ENSG00000146670
CDCA8	ENSG00000134690
CDK1	ENSG00000170312
CDK6	ENSG00000105810
CDKN2C	ENSG00000123080
CDT1	ENSG00000167513
CENPM	ENSG00000100162
CENPN	ENSG00000166451
GINS2	ENSG00000131153
H2AC14	ENSG00000276368
H2AFX	ENSG00000188486
H3C10	ENSG00000278828
H3C12	ENSG00000197153
H3C15	ENSG00000203852
H3C4	ENSG00000197409
HIST2H3D	ENSG00000183598
KIF2C	ENSG00000142945
LMNB1	ENSG00000113368
MLF1IP	ENSG00000151725
NDC80	ENSG00000080986
PCNA	ENSG00000132646
PTTG1	ENSG00000164611
SMC4	ENSG00000113810

Supplementary Figures

Figure S1. Study consort diagram, related to Figure 1 and to sample selection in case-control study design in STAR methods.

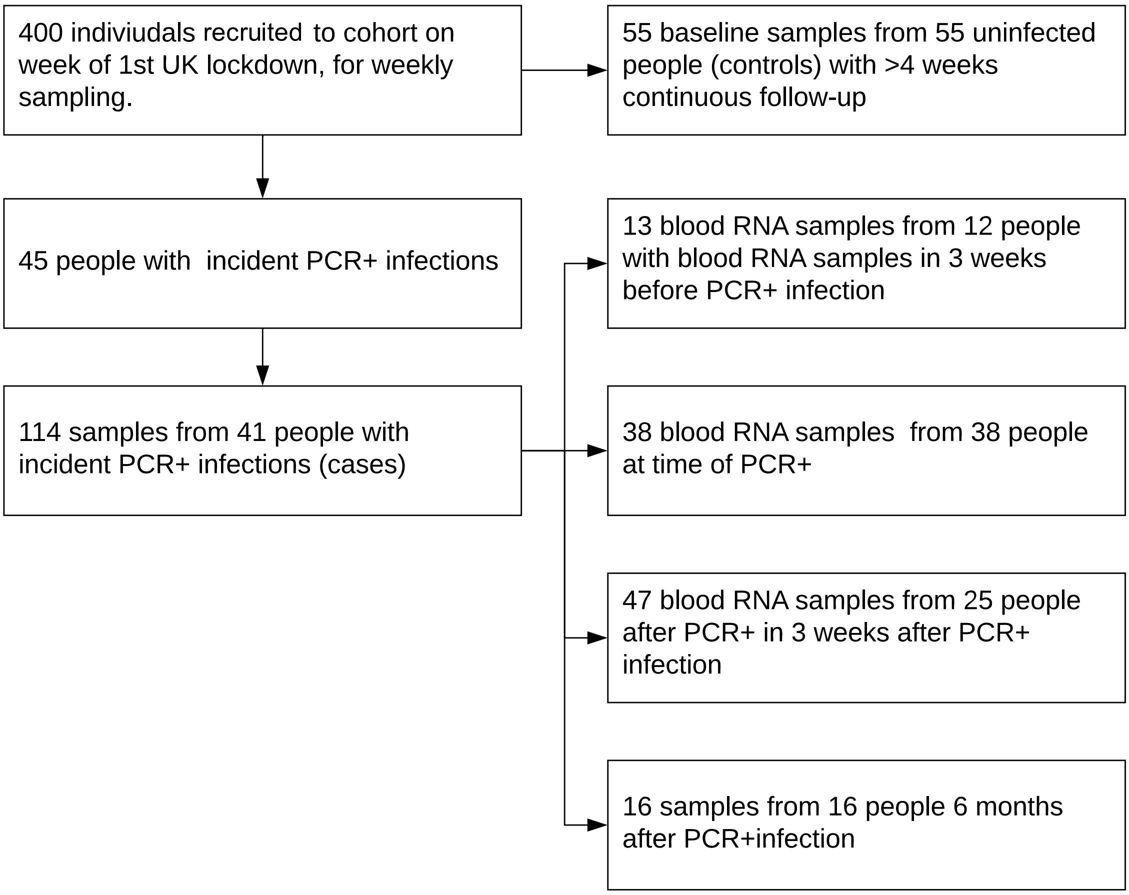

Figure S2. Batch correction, molecular degree of perturbation and identification of co-regulated modules in blood transcriptomic data, related to analysis of Blood RNA sequencing data in STAR methods, and Figure 1.

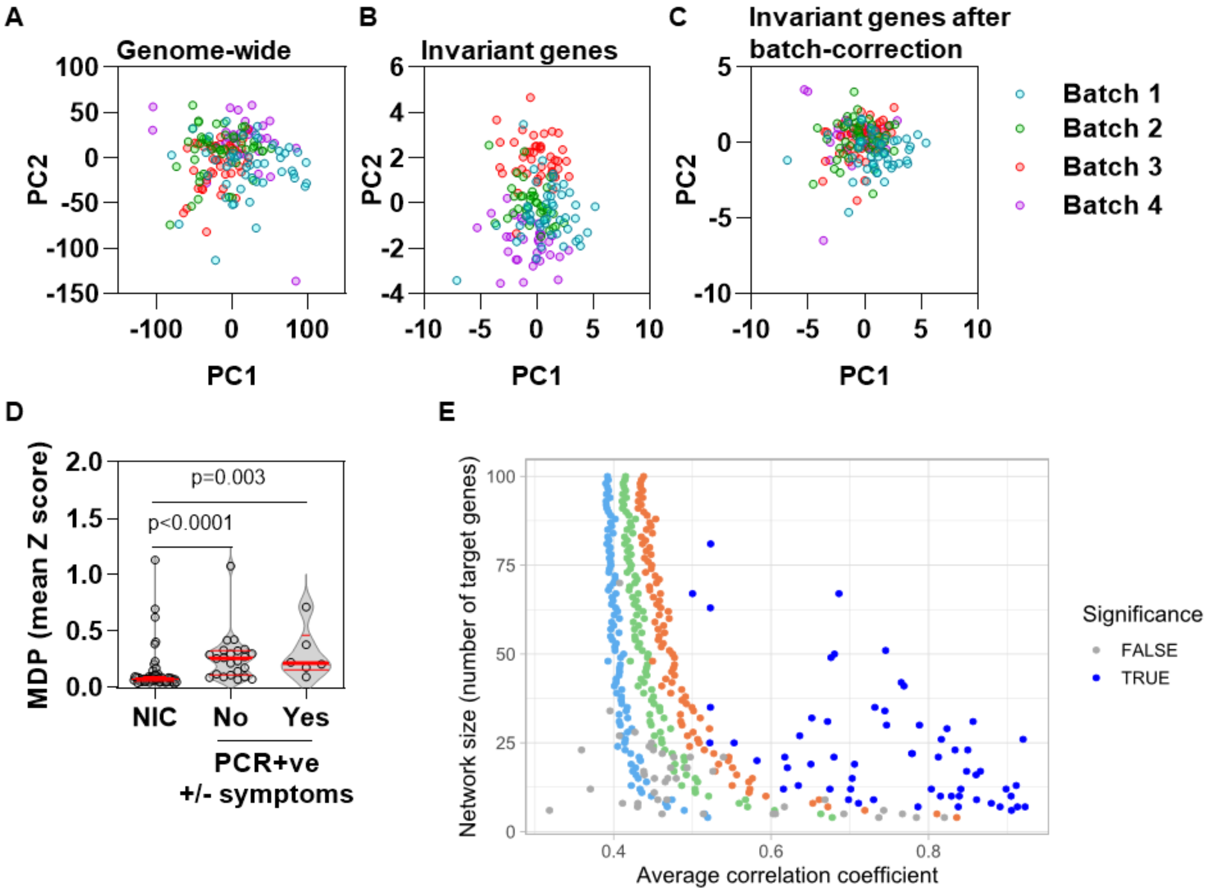

(A) Principal component analysis for genome-wide data, **(B)** 10% least variant genes from blood transcriptional profiles stratified by sample processing batch, and **(C)** for least variant genes after batch correction. **(D)** Molecular degree of perturbation (MDP) in blood transcriptomes for each individual expressed as the mean of genome-wide standard deviations (Z scores) >2 from the mean of non-infection controls (NIC), among samples from non-infection controls and samples from individuals with co-incident PCR+ve infection with (Yes) or without (No) case-defining symptoms. Individual data points shown with violin plots depicting median, IQR and frequency distributions. P values derived from Mann-Whitney tests (ns=not significant). **(E)** Figure 2, and blood RNA sequencing analysis in STAR methods. Z-scores derived from the distribution of average correlation coefficients obtained from 100 iterations of randomly selecting groups of genes from blood transcriptomic data are shown in light blue (z-score=1), green (z-score=2) and orange (z-score=3). Average correlation coefficients of molecular networks associated with upstream regulators of differentially expressed genes between individuals with incident SARS-CoV-2 infection and noninfection controls are shown in dark blue (z-score ≥2, false discovery rate ≤0.05) and grey (z-score ≤2 +/- FDR ≥0.05) compared to equivalent sized random gene networks.

Figure S3. Bioinformatic analysis of modules derived from upstream regulator analysis of differentially expressed genes in blood transcriptome associated with SARS-CoV-2 infection, related to Figure 1, Figure 2 and Blood RNA sequencing analysis in STAR methods.

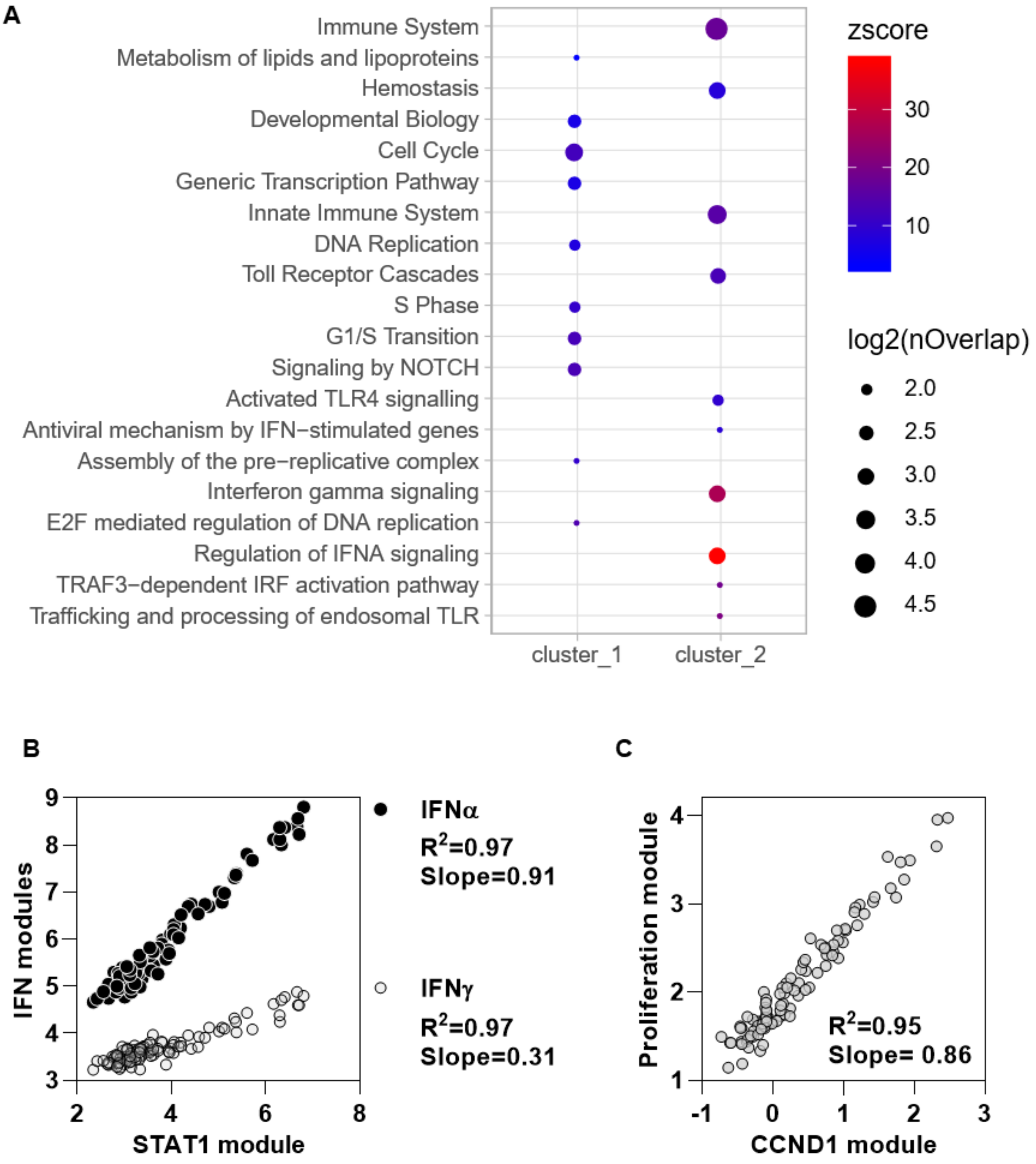

(A) Enrichment of Reactome pathways among the two clusters of predicted upstream regulators associated with co-incident infection (depicted main manuscript Figure 1 and listed Supplementary Data File 1). Node size represents number of upstream regulators associated with each pathway and statistical enrichment represented by Z score. **(B)** Correlation of STAT1-regulated transcriptional module with interferon (IFN)α and IFNγ modules, and **(C)** correlation of CCND1-regulated transcriptional module with independently derived proliferation module in all time points (-3 to +3 weeks) from individuals with SARS-CoV-2 infection.

Figure S4. STAT1 and CCND1 regulated module expression over time in blood transcriptomic data from individuals experimentally infected with acute respiratory viruses, related to Figure 2.

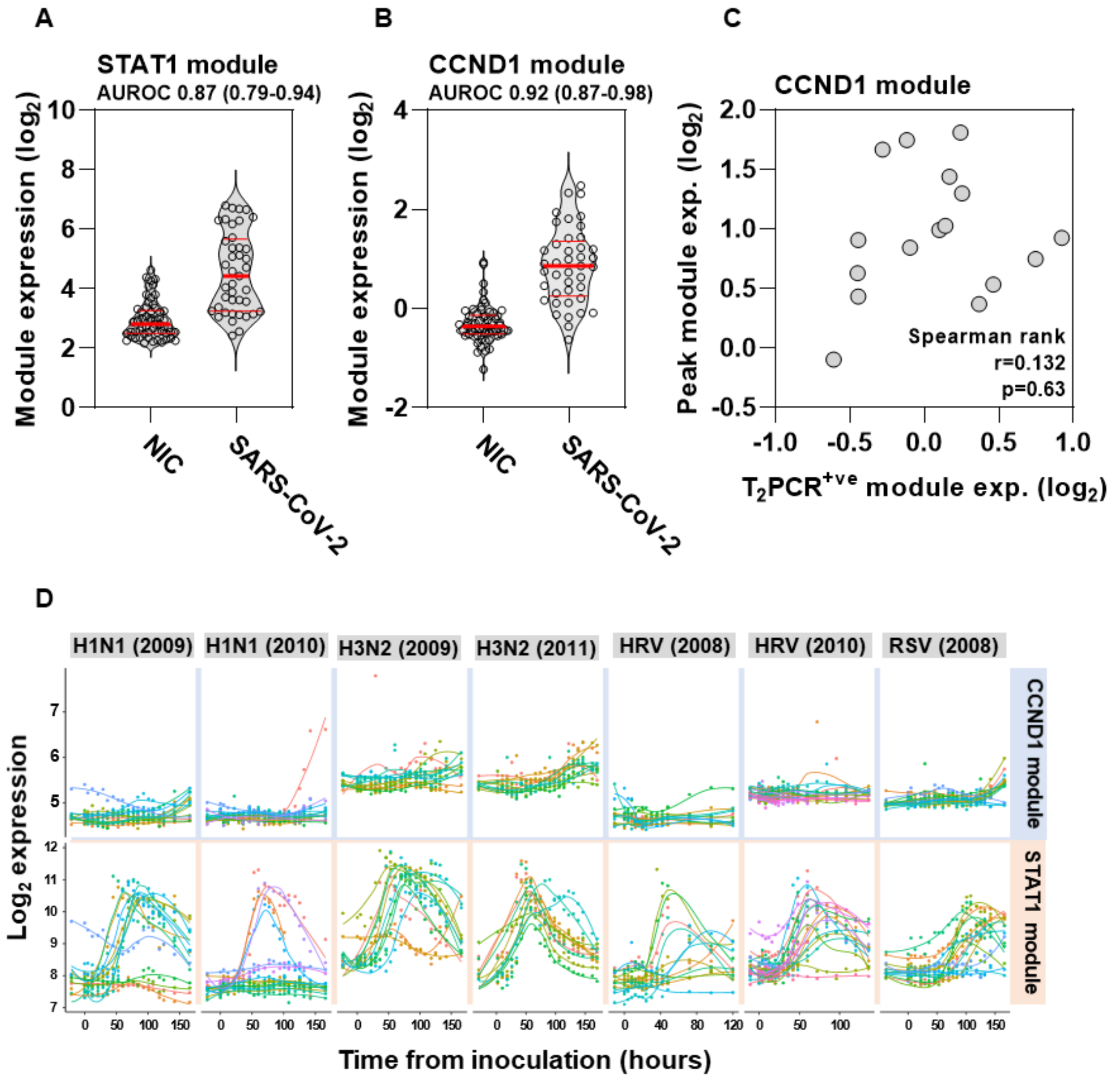

(A-B) Comparison of peak STAT-1 and CCND1-regulated module expression in samples from individuals with SARS-CoV-2 infection and non-infection controls showing area under the receiver operating curve ±95% confidence intervals for these modules to discriminate between the two groups. **(C)** Correlation of peak CCND1-regulated module expression in samples from individuals with SARS-CoV-2 infection with CCND1-regulated module expression 2 weeks after first positive PCR test. **(D)** Expression scores for STAT1 and CCND1 modules in publicly available data from seven (non-SARS-CoV-2) human viral challenge studies (GSE73072). Analysis restricted to participants with evidence of infection following inoculation, as per original study definition (total n=92 participants). Colours represent individual participants with longitudinal sampling. Each column represents a different challenge study, with the virus and study year shown in the column headers. HRV = human rhinovirus; RSV = respiratory syncytial virus.

Figure S5. Multiparametric flow cytometry of PBMC and frequency of selected lymphocytes and their activation/proliferative state, related to Figure 3.

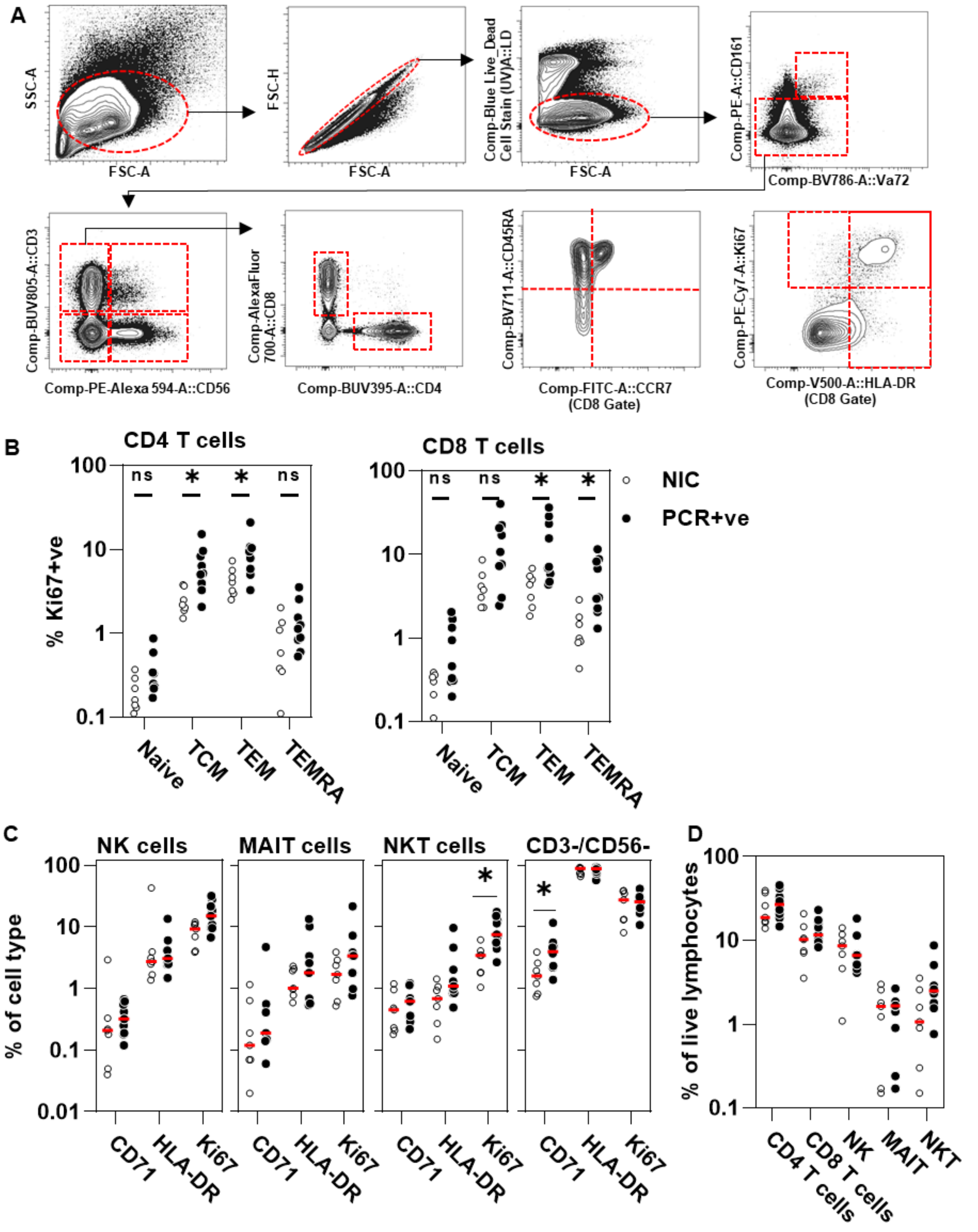

(A) Example gating strategy for flow cytometric data showing gating for; lymphocytes, singlets, live cells (fixable live dead-), MAIT cells (CD161++ TCR Va7.2+) and non-MAIT cells, CD3+CD56- T cells, CD3+CD56+ NKT cells, CD3-CD56- lymphocytes, CD3-CD56+ NK cells, CD4+ and CD8+ T cells, CD45RA vs. CCR7 co-staining, and HLA-DR vs. Ki67 co-staining. **(B)** Frequency of CD4+ and CD8+ T cell subsets, **(C)** non-T cell lymphocyte subsets expressing selected activation markers (CD71 and HLA-DR) and the cell proliferation marker (Ki67), and **(D)** comparative frequency of selected lymphocyte subsets among participants with co-incident infection (PCR+) and non-infection controls (NIC). Data points with median values (red). (*p<0.05 by Mann-Whitney Test for each group compared to NIC).

Figure S6. Identification of expanded TCRs, expansion of TCR beta chain sequences and abundance of MAIT/iNKT cell sequences in SARS-CoV-2 infection, related to Figure 4 and analysis of blood TCR sequencing data in STAR methods.

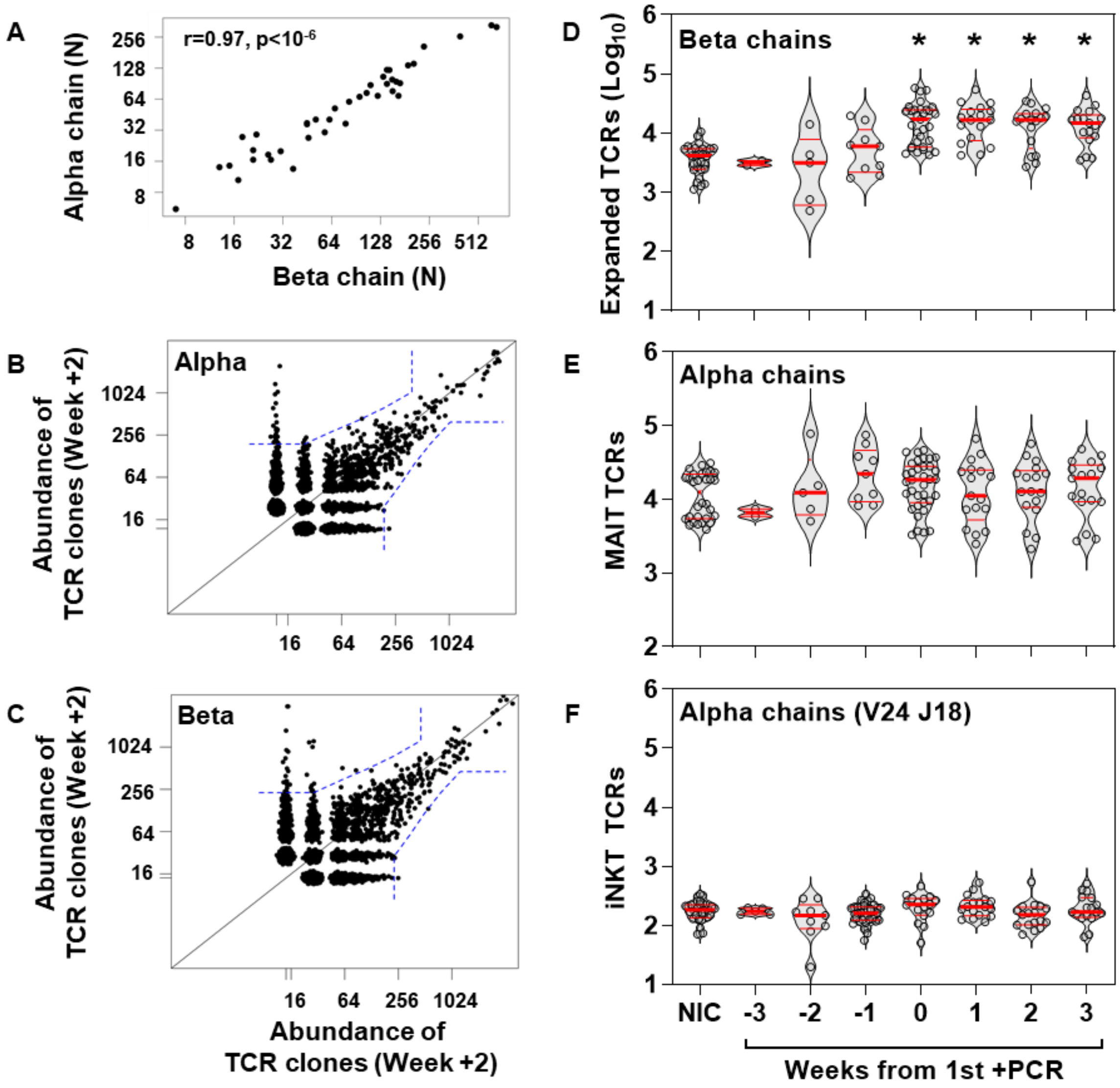

(A) Correlation of numbers of alpha and beta chain TCR sequences per sample. **(B)** Representative examples of identification of statistically expanded alpha, and beta chain TCR sequences by comparison of abundance of individual TCR sequences between two time points (blue dashed boundaries represent the thresholds for abundance of TCR sequences with false discovery rate of <1 in 1000) **(C)** Enumeration of expanded TCR alpha chain sequences (per million total sequences) in non-infection controls (NIC) and samples from infected individuals stratified by time from first positive PCR. **(D)** Abundance of MAIT and **(E)** iNKT cell associated alpha chain sequence in non-infection controls and samples from infected individuals stratified by time from first positive PCR. Individual data points shown with violin plots depicting median, IQR and frequency distributions. (*FDR<0.05 by Kruskal-Wallis Test for each group compared to NIC.

Figure S7. Ex vivo T cell responses to structural and non-structural peptides 16 weeks post-infection, related to Figure 4 and analysis of blood TCR sequencing data in STAR methods.

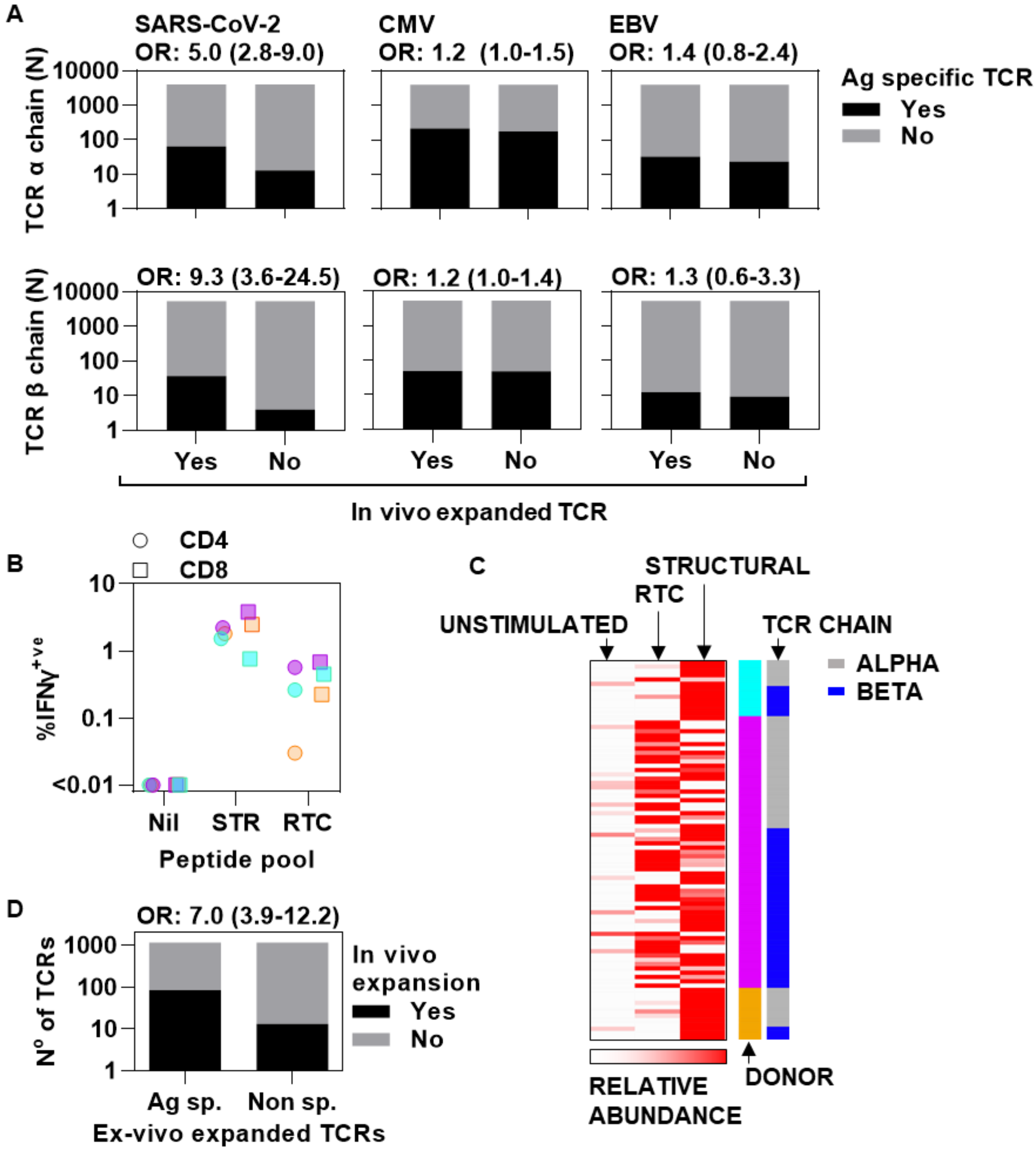

(A) Number of antigen-specific TCR sequences (alpha and beta-chains) for SARS-CoV-2, cytomegalovirus (CMV) and Epstein-Barr Virus (EBV) among expanded TCR sequences in all time points (-3 to +3 weeks) from individuals with SARS-CoV-2 infection and among non-expanded TCRs from the same samples, giving the odds ratio (±95% confidence interval, Fisher's exact test) for enrichment of antigen specific TCR sequences in each case. **(B)** Frequency of interferon (IFN)γ producing CD4 and CD8 T cells in PBMC (N=3 individuals stratified by colour of data points) following 8 days culture in the presence of IL2 and soluble anti-CD28 ±stimulation with overlapping peptides covering structural (STR) or replication and transcription complex (RTC) proteins. **(C)** Relative abundance of TCR alpha and beta chains from the experiments in (A) that coincide with expanded sequences in peripheral blood of the same individuals at the time of incident PCR positive infection. **(D)** Overlap of in vivo expanded TCR sequences (alpha and beta-chains) associated with incident PCR positive SARS-CoV-2 infection with ex vivo expanded TCRs SARS-CoV-2 peptide stimulated and unstimulated PBMC giving the odds ratio (±95% confidence interval, Fisher's exact test) for enrichment of virus specific TCR sequences.
